# Diversity and Distribution of Phenol Oxidase Producing Fungi from Soda Lake and Description of *Curvularia lonarensis* sp. nov.

**DOI:** 10.3389/fmicb.2016.01847

**Published:** 2016-11-22

**Authors:** Rahul Sharma, Om Prakash, Mahesh S. Sonawane, Yogesh Nimonkar, Priyanka B. Golellu, Rohit Sharma

**Affiliations:** ^1^Microbial Culture Collection, National Centre for Cell SciencePune, India; ^2^Institute of Bioinformatics and Biotechnology, Savitribai Phule Pune UniversityPune, India

**Keywords:** *Curvularia*, fungal diversity, extremophilic fungi, lonar lake, phenol oxidase, soda lake

## Abstract

Soda lake is hyper alkaline and saline habitat located in closed craters with high evaporation rate. In current study fungal diversity from water and sediment samples of a soda lake (Lonar lake) located in Buldhana district of Maharashtra, India was investigated using extensive culturomics approach and mimicking the natural conditions of Lonar lake in culture media. A total of 104 diverse isolates of extremophilic fungi were recovered from this study and phylogenetically characterized by internal transcribed spacer (ITS) region sequencing. In addition, due to important role of phenol oxidase, and peroxidase in degradation of toxic phenol, lignin, etc., all isolated pure cultures were also screened for extracellular phenol oxidase and peroxidase production potential. Diversity analysis indicated that different groups of extremophilic fungi are present in the water and sediment samples of Lonar lake. A total of 38 species of fungi belonging to 18-different genera were recovered. Out of 104 isolates 32 showed ≤97% sequences similarity, which were morphologically different and could be potential novel isolates of extremophilic fungi. However, out of 104 isolates only 14 showed the extracellular phenol oxidase production potentials at alkaline pH. *Curvularia* sp. strain MEF018 showed highest phenol oxidase production at alkaline condition and had low sequence similarity with previously characterized species (96% with *Curvularia pseudorobusta*). Taxonomic characterization (morphological and physiological) and multi locus sequence analysis (MLSA) using combined alignment of ITS-LSU-*gpd* of strain MEF018 showed that it is a novel species of the genus *Curvularia* and hence proposed as *Curvularia lonarensis* sp. nov.

## Introduction

Due to immense biotechnological applications of extremophilic enzymes, study of microbial diversity of extreme habitats like soda lake, hot springs, Arctic, and Antarctic polar regions, acid mine drainage and thermal vents are of current interest among the microbiologists (Vargas et al., 2004; Jayani et al., 2005; Calvez et al., 2009; Das et al., 2009; Burgaud et al., 2010; Brown et al., 2015; Chaput et al., 2015). Similar to Bacteria and Archaea, fungi as saprophytes or mutualistic symbionts also provide valuable ecosystem services including degradation of organic materials and mineralization and mobilization of nutrients. Therefore, cultivation and characterization of novel extremophilic fungi from unusual habitats and study of their physiology, genetics and biotechnological potential are equally important from ecological and industrial perspective. According to Ostergaard and Olsen (2011), 75% of the industrial enzymes come from only five genera of fungi which reveal that maximum fungi remain industrially unutilized or un-explored. Moreover, novel fungi are not explored for their potential uses. Despite the immense importance of fungal enzymes in biogeochemical cycling of materials and industrial applications, unlike bacteria little attention has been given on cultivation and characterization of extremophilic fungi from extreme habitats like soda lakes. Although, fungi prefer acidic to neutral pH range for growth, reports on alkaliphilic, and halophilic fungal species from soda lakes like Magadi lake of Kenya, Natron lake of Tanzania (pH 11–12), and Dead sea of Israel are available in literature (Oren and Gunde-Cimerman, 2012; Grum-Grzhimaylo et al., 2013a,b, 2016). These relatively recent contributions are important in light of scarcity of similar studies on fungi and potential of fungi isolated from these unusual habitats.

Phenol oxidase and peroxidase are the enzymes which are important in lignin degradation, humification, carbon mineralization, and dissolved carbon export (Sinsabaugh, 2010). In fungi polyphenol oxidases, particularly laccases play role in lignin degradation, fungal spore formation, pigmentation, detoxification of toxic compounds, pathogenesis, and fungal morphogenesis. Phenol oxidase also play important role in depletion and accumulation of soil organic matter. It has been observed that level of soil organic matter decrease with increasing activity of phenol oxidase, while low level of phenol oxidase promotes its accumulation in soil. In addition, it also degrades toxic phenolic compounds, protects microbial cells from their harmful effects, and play an important role in management of plant residue with high lignin content. Phenol oxidases have widespread applications in pollutant degradation, effluent decolouration, pulp bleaching, removal of phenolics from wines, oxidation of dye, enzymatic conversion of chemical intermediates, biofuel production, etc., Most of the polluted habitats like industrial effluents, leachates, hospital wastes etc. show extreme condition in one or the other aspects. Therefore, organism with potential to produce enzyme in extreme condition has special significance for enzymatic bioremediation of pollutants in comparison to their mesophilic counterparts. Hence, considering the importance of phenol oxidase in biotechnology and carbon cycling of natural ecosystem we screened isolated strains for phenol oxidase production. The chemistry, function and biotechnological use of laccases have recently been reviewed (Baldrian, 2006).

In the current study we cultivated wide range of alkaliphilic and halophilic fungi from water and sediment samples of hyperalkaline and saline Lonar lake using extensive culturomic approaches i.e., use of different media and culture conditions. In addition, we also screened all the isolated fungi for production of phenol oxidase and peroxidase in alkaline condition. Identification of all isolates was done by sequencing internal transcribed spacer (ITS) region. Finally, we did the taxonomical characterization of a novel species of alkaliphilic and halophilic *Curvularia* which showed efficient phenol oxidase production potential at alkaline pH and proposed it as *Curvularia lonarensis* sp. nov. Multi locus sequence analysis (MLSA) was used to ascertain its position within the genus *Curvularia*. To the best of our knowledge, this is the first report on study of fungal diversity and their phenol oxidase producing potential from Lonar lake.

## Materials and methods

### Collection of samples and geochemical characterization of sampling site

**Figure 1:**
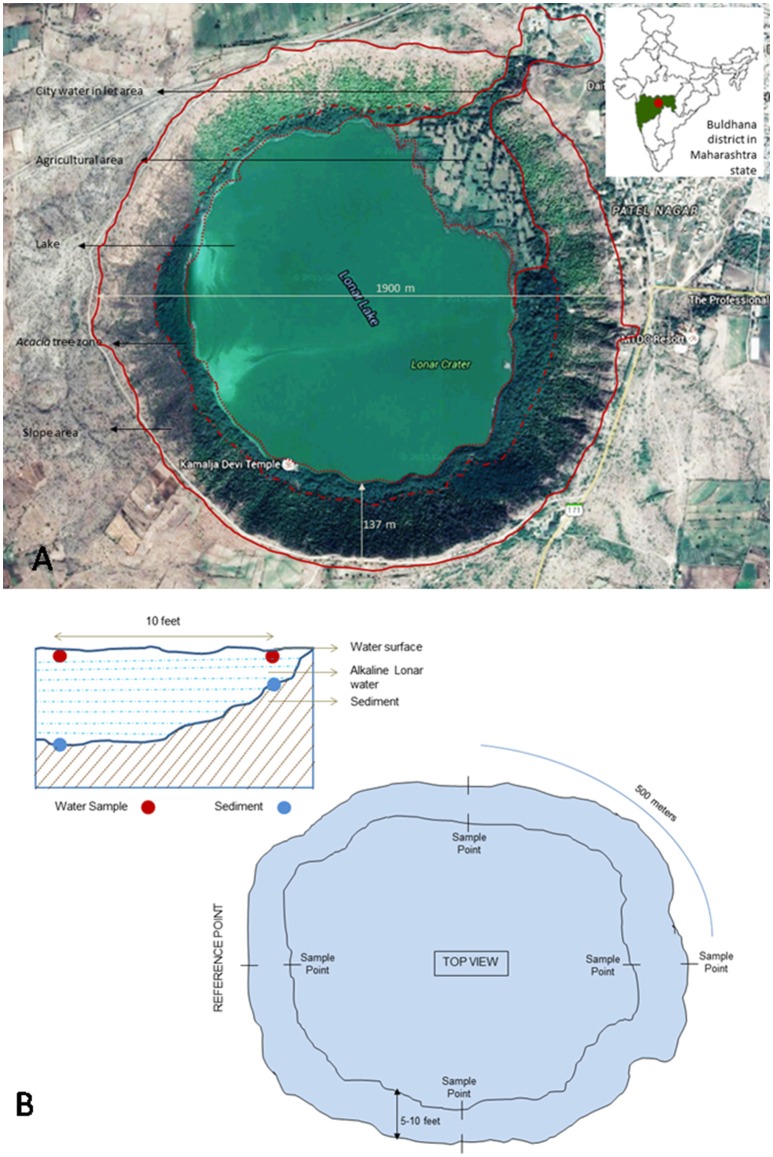
**(A)** Google image of Lonar lake and its location on Indian map (modified from google maps) (Map data: Google, DigitalGlobe). **(B)** Schematic representation of lake and details of sampling strategy.

Lonar lake is a hyper-saline and alkaline soda lake located in Buldhana district of Maharashtra, India (19°58′36″N 76°30′30″E) and created by meteor impact during Pleistocene Epoch. The diameter of lake is approximately 1.8 km with 135 m of slope. Due to its unique ecological and geological features it is a site of interest to microbiologists for cultivation of extremophilic microbes. Photographic and tabular representation of lake and its geochemistry are presented in Figure 1A, Table 1 respectively. In current study sediment and water samples were collected from Lonar lake for cultivation of alkaliphilic and halophilic fungi. Water and sediment samples were collected in 50 ml capacity pre-sterilized Falcon tubes (Falcon, USA). Sampling was done from four different collection points located at equal distance (500 m) on circumference of the lake from a reference point. Samples were collected from shore line as well as from 3 m inside the lake from the periphery with a water column depth of approximately 30 cm. Three samples were collected from each point and mixed to form a compound sample. Thus, a total 16 compound samples of water and sediment were collected. Diagram of sampling strategy is given in Figure 1B. Collected samples were stored on crushed ice and transferred to the laboratory and stored at 4°C until further processing.

**Table 1:** **Quantification of chemical parameters of Lonar lake sediments (figures in bold shows values of important parameters)**.

**S. no**.	**Chemical parameters**	**Chemical parameters (% w/w)**
1	Total Dissolved Solids @ 105°C (TDS)	1.0374
2	Total Organic Carbon (TOC)	0.2527
3	Total Kjeldhal Nitrogen (TKN)	0.193
4	Total Phosphorus (P) as ${\text{PO}}_{4}^{3-}$	3.0628
5	Nitrates (${\text{NO}}_{3}^{-}$)	0.7913
6	**Sodium as NaCl**	**1.70489**
7	Carbonates (${\text{CO}}_{3}^{-}$)	0.2295
8	Chlorides (Cl^−^)	0.2093
9	Ammonia (NH_3_)	0.0253
10	Sulfates (${\text{SO}}_{4}^{2-}$)	0.0533
11	**Calcium as (Ca)**	**0.5161**
12	Cobalt as Co	0.001964
13	Nickel (Ni)	0.000941
14	Boron as B	0.00123
15	**Magnesium as Mg**	**1.0992**
16	Potassium as K	0.0479
17	**Iron as Fe**	**2.1934**
18	Copper as Cu	0.0053

*Data taken from Antony et al. (2010)*.

### Isolation of fungi

In order to isolate diverse range of fungi, an exhaustive culturomics approach with wide range of culturing conditions (culture media and temperature) were used. To mimic the natural conditions of salt and pH all the media used for isolation were prepared in Lonar lake water and adjusted to pH 10. Serial dilutions (10^−1^–10^−10^) of sediment and water were prepared in filter sterilized Lonar lake water and 100 μl of serially diluted sediment and water samples were plated on the surface of different fungal media. Based on the growth and diversity of fungi captured during preliminary screening on 32-different media (available in the laboratory), only 9 media viz., PDA, CDA, HK Medium 8A, HK Medium 13A, HK Medium 16A, HK Medium 19A, HK Medium 21A, HK Medium 22A, HK Medium 28A (Hi-Media, India) were selected for actual isolation purpose. The compositions of different HK media used for isolation purpose are given in Table ST1. Plates were incubated at two temperatures 28 ± 1°C and 35 ± 1°C up to 21 days. Morphologically different colonies were picked and purified on potato dextrose agar (PDA) plates (pH 10) using hyphal tiping method from the margin of actively growing colonies. After purification, basic morphological characteristics of the isolated fungi were recorded. Purified fungal colonies were preserved with 10% glycerol in liquid nitrogen (−196°C) and in deep freezer (−80°C) at Microbial Culture Collection division of National Centre for Cell Science, Pune India as discussed in Prakash et al. (2013).

### Genomic DNA extraction and internal transcribed spacer (ITS) region amplification

Genomic DNA was extracted using the CTAB method as discussed in Voigt et al. (1999). In brief, small amount of fungal mycelia was suspended in 200 μl of extraction buffer containing 10 μl of proteinase K, 5 μl- β-mercaptoethanol, and glass beads in Eppendorf tube and crushed by pestle. After that, 300 μl extraction buffer was added again and vortexed for 5 min. Content was incubated for 2 h at 60°C on a dry bath. After incubation, content was mixed with 140 μl of 5 M NaCl and 64 μl of 10% CTAB and vortexed for 5 min, and incubated at 65°C for 1 h. Total content was mixed with equal volume of chloroform: isoamyl alcohol and incubated for 30 min on ice. Post precipitation, content was centrifuged at 10,000 rpm for 10 min, and aqueous phase was transferred in a fresh eppendorf tube. DNA was precipitated with 1/10th volume of 3 M sodium and washed with 70% ethanol, air dried, and suspended in 100 μl of Tris EDTA buffer. Quantity and quality of DNA was checked by NanoDrop spectrophotometer (Thermo Scientific, USA) and 1% agarose gel electrophoresis.

Fungal ITS region was amplified using universal primers ITS1 (5′-TCCGTAGGTGAACCTGCGG-3′) and ITS4 (word5′-TCCTCCGCTTATTGATATGC-3′) as discussed in White et al. (1990). The D1/D2 region of large subunit (LSU) was amplified using primers LROR (word5′-ACCCGCTGAACTTAAGC-3′) and LR5 (5′-TCCTGAGGGAAACTTCG-3′) as per Vilgalys and Hester (1990). Standard PCR protocol and PCR cycle parameters were used for both. However, the glyceraldehyde-3-phosphate dehydrogenase (*gpd*) gene was amplified by primer set *gpd1* (word5′-CAACGGCTTCGGTCGCATTG-3′) and *gpd2* (5′-GCCAAGCAGTTGGTTGTGC-3′) as per the PCR conditions given by Berbee et al. (1999). PCR reaction was performed using 2720 thermal cycler (Applied Biosystems, US). Amplified product was checked on 1.2% agarose gel and purified by PEG- NaCl method as mentioned in Sambrook et al. (1989).

### Molecular identification and phylogenetic study

Purified PCR products were sequenced using the ABI BigDye Terminator Cycle Sequencing Ready Reaction kit (Applied Biosystems, Foster City, CA) as per manufacturer's instructions and ABI 3730xl (Applied Biosystem, USA) automated DNA sequencer. The quality of the raw sequences were checked and edited using software ChromasPro version 1.34 (Technelysium Pvt. Ltd., Tewantin, Queensland, Australia) and Sequence Scanner version 1.0 (Applied Biosystems, US). Similarity search was carried by BLASTn search with the available ITS sequences in GenBank database (Zhang et al., 2000). Taxonomic affiliations for known species were obtained by a threshold cut-off of ~97% using ITS sequences (Blaalid et al., 2013). The ITS sequences showing <97% similarity were considered as belonging to probable novel taxa. For phylogenetic analysis, published sequences of closely related organisms were retrieved in FASTA format and aligned in *CLUSTAL*-W. Sequence alignment and phylogenetic analysis was performed with *MEGA* v.5 computer program (Tamura et al., 2011). For MEF018, the assembled consensus sequences of ITS, LSU, and *gpd* gene were aligned separately in *CLUSTAL*-W using *MEGA* v.5. A concatenated alignment of three regions ITS-LSU-*gpd* was generated using MEGA v.5 and phylogenetic analysis was conducted using maximum likelihood (ML) and accuracy of the methods was assessed using 1000- bootstrap replicates. Evolutionary distances were computed using the Kimura 2-parameter method (Kimura, 1980) and are in the units of the number of base substitutions per site. Bootstrap confidence intervals were set at 50% (Saitou and Nei, 1987). The neighbor joining (NJ) and maximum parsimony (MP) analysis was also done which yielded similar topologies. All ITS region rRNA gene sequences generated from this study were submitted in NCBI GenBank database under accession numbers KT315397 - KT315422, KT315424 - KT315430, KT315432 - KT315451, KT315453 - KT315503. The D1/D2 region sequence of LSU and *gpd* gene sequence has been submitted to NCBI [KY007019 (*gpd*); KY007018 (LSU)]. The GenBank accession numbers strain ID or culture collection numbers and source of the isolates used in the phylogenetic study of genus *Curvularia* is compiled in Table ST2.

### Screening of strains for phenol oxidase production

All the selected strains were screened for extracellular phenol oxidase production using ABTS [2, 2′-azino-bis (3-ethylbenzothiazoline-6-sulphonic acid)] as substrate (Floch et al., 2007). For that, PDA medium was supplemented with 10 mM ABTS, sterilized by autoclaving and poured into Petri plates. After solidification, 5 mm diameter fungal bits were cut from actively growing pure cultures of isolated fungi using sterile Cork-borer and inoculated in the center of the plates. Plates were incubated in the dark and observed for the development of colored zone around fungal colonies (Figure S1). Colonies showing positive result for phenol oxidase production were selected for comparative study. Efficiency of the extracellular phenol oxidase production was determined using the phenol oxidase assay as discussed previously (Floch et al., 2007; Sinsabaugh, 2010). In brief, pure culture of selected fungi were raised in liquid medium and amount of phenol oxidase (unit of enzyme production / ml culture broth) was assayed at different time point using the culture broth as crude source of extracellular phenol oxidase and ABTS as substrate. Optima and range of growth at different temperatures, pH and NaCl concentrations of phenol oxidase producing fungi were tested as discussed in Lin et al. (2012). In addition the range and optima of enzyme production at different pH and temperatures was also evaluated by inoculating equal amount of inoculum in optimum medium with different pH and incubation at different temperatures. To determine the correlation between mycelial biomass and enzyme production, mycelial dry weight was also recorded. For that equal amount of fungal bits were inoculated in Erlenmeyer flask in replicate of three. Mycelia was harvested at different time interval on pre-weighted Whatman® Cellulose Filter Paper and incubated at 60°C for 24 h. Cellular biomass was recorded by reducing the weight of filter paper.

### Taxonomic characterization of *Curvularia lonarenesis* sp. nov. (morphological and physiological study)

Macro-morphological characters like colony morphology, sporulation pattern, surface structure, type, and rate of mycelial growth, colony size, margin, pigmentation, zonation, exudation under different media, and temperature conditions were recorded as discussed in Sharma et al. (2015). Micro-morphological features were observed by mounting the fungus on lactophenol-cotton blue (Hi-Media, India) and observing under light microscope Olympus BX53 (Olympus, Japan). Photomicrographs were taken by ProgRes C5 camera (Jenoptik, USA) attached to the microscope. For Differential Interference Contrast (DIC) microscopy, the slide was observed and images captured on a fully automated upright fluorescence microscope coupled with monochrome and color CCD cameras (Olympus, Japan). DIC was performed at Indian Institute of Science Education and Research, Pune, India. In-depth taxonomical characterization of *Curvularia* sp. strain MEF018 was conducted as discussed in Madrid et al. (2014). Optimal growth medium was tested by screening strain MEF018 on different range of fungal media like, oat meal agar (OA), Saboraud's dextrose agar (SDA), malt extract agar (MEA), potato dextrose agar (PDA), and Czapek dextrose agar (CDA). The best medium for growth was then subjected to pH study (5–14). After keeping both these factors standard, temperature range was tested (5–40°C). As the salinity of Lonar lake is 3%, the salt tolerance was checked by growing the fungus on a range of salt concentrations (3–12%).

## Results

**Figure 2:**
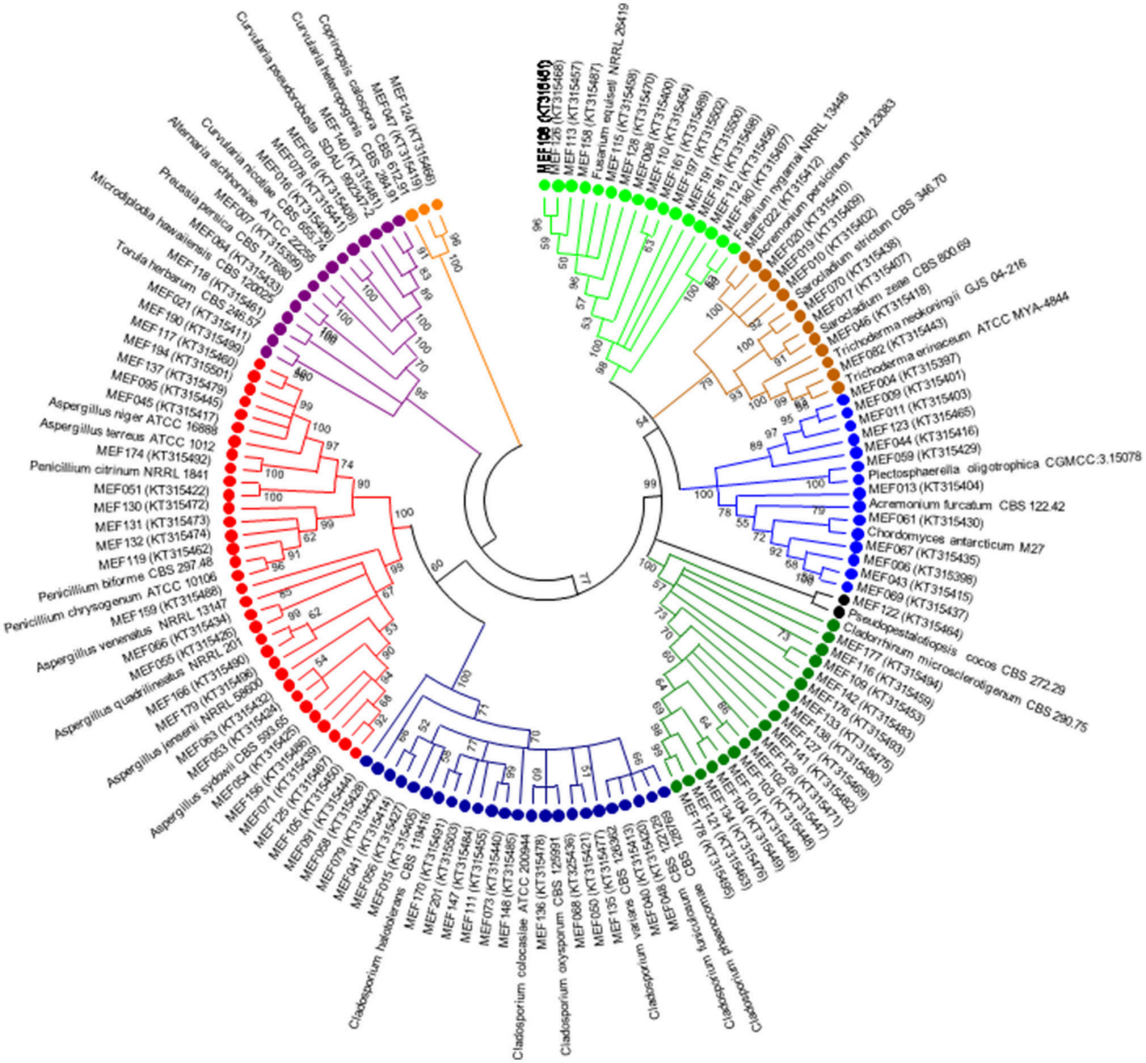
**Phylogenetic tree constructed using ITS sequences of 104 isolated strains of fungi along with homologous sequences from type strains of closest match in GenBank**. The evolutionary history was inferred using the neighbor-joining method. The optimal tree with the sum of branch length = 3.96668506 is shown. The evolutionary distances were computed using the Kimura 2-parameter method (Kimura, 1980) and are in the units of the number of base substitutions per site. The analysis involved 139 nucleotide sequences. There were a total of 659 positions in the final dataset. Evolutionary analyses were conducted in MEGA5 (Tamura et al., 2011). (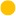 *Psathyrellaceae*; 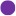 *Pleosporaceae*; 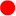 *Trichocomaceae*; 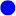 *Davidiellaceae*; 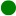 *Lasiosphaeriaceae* 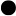 *Amphisphaeriaceae*; 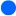 *Plectosphaerellaceae*; 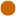 *Hypocreaceae*; 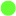 *Nectriaceae*).

A total of 104 isolates from different groups of fungi were cultivated from water and sediment samples of Lonar lake. Data related with percent identity and query coverage based on ITS sequences (GenBank database) along with key features of closely related fungal strains are presented in Table 2. Sequence similarity data indicated that isolates from Lonar lake showed similarity with members of 18 genera and 38 different fungal species. Except 2 isolates (*Coprinopsis calospora* of *Basidiomycota* belonged to family *Psathyrellaceae*), all other strains belonged to members of phylum *Ascomycota*. Diversity data from this study indicated that the closest relatives of our isolates showed similarity with fungal species isolated from diverse habitats and most of them are associated with alkaline and saline habitats or soda lakes (Table 2). Phylogenetic analysis using sequences from ITS and 5.8 S region of rRNA gene showed 8 ascomycetous lineages and one basidiomycetous lineage (Figure 2). In total 32 isolates from different genera including *Cladorrhinum, Cladosporium*, and others showed ≤97% sequence similarity with previously described fungal species and are potential novel genera or species according to the sequence based species delineation in fungal taxonomy (Blaalid et al., 2013). In addition, phylogenetic study also represented two separate clades belonging to genera *Cladosporium* and *Cladorrhinum* which contained large number of isolates. Four isolates belonged to genus *Curvularia*, but none of them showed close relatedness with existing members which indicated their taxonomical novelty.

**Table 2:** **List of all the isolated fungi from Lonar lake with their closest relative in GenBank database and their specific feature reported in literature (figures in bold shows less sequence similarity of probable novel isolates)**.

**Strain no. (MEF xxx)**	**% similarity with closest match in genbank**	**Query coverage**	**Max identity**	**Site of isolation**	**Specific feature**
MEF004 (KT315397)	*Chordomyces antarcticum* M27 (KJ443241)	95	**95**	Alkaline soil near Karakul lake, Russia	NA
MEF006 (KT315398)	*Chordomyces antarcticum* M27 (KJ443241)	94	99	Alkaline soil near Karakul lake, Russia	NA
MEF007 (KT315399)	*Alternaria eichhorniae* ATCC 22255 (NR_111832)	100	99	Water hyacinth, *Eichhornia crassipes*, India	Pathogenic to water-hyacinth
MEF008 (KT315400)	*Fusarium equiseti* NRRL 26419 (NR_121457)	96	99	Soil, Braunschweig, Germany	Mycotoxin production
MEF009 (KT315401)	*Chordomyces antarcticum* M27 (KJ443241)	94	**95**	Soil, China (using low carbon medium)	NA
MEF010 (KT315402)	*Acremonium persicinum* JCM 23083 (NR_131260)	100	99	Coastal sand under *Ammophila arenaria*, France	Isolation of Acremine and Heptapeptides
MEF011 (KT315403)	*Chordomyces antarcticum* M27 (KJ443241)	94	**95**	Alkaline soil near Karakul lake, Russia	NA
MEF013 (KT315404)	*Chordomyces antarcticum* M27 (KJ443241)	94	99	Alkaline soil near Karakul lake, Russia	NA
MEF015 (KT315405)	*Cladosporium phaenocomae* CBS 128769 (NR_119950)	98	99	Flower of *Phaenocoma prolifera*, Western Cape Province, South Africa	NA
MEF016 (KT315406)	*Curvularia nicotiae* CBS 655.74 (KJ909772)	90	**96**	Soil, China (using low carbon medium)	NA
MEF017 (KT315407)	*Sarocladium zeae* CBS 800.69 (NR_130685)	100	99	Stalk, *Zea mays*, Nebraska	NA
MEF018 (KT315408)	*Curvularia pseudorobusta* HSAUP 992347 (NR_130653)	100	**96**	An undetermined plant of Poaceae, Guangxi, Beihai, China	NA
MEF019 (KT315409)	*Acremonium persicinum* JCM 23083 (NR_131260)	100	99	Coastal sand under *Ammophila arenaria*, France	Isolation of Acremine and Heptapeptides
MEF020 (KT315410)	*Acremonium persicinum* JCM 23083 (NR_131260)	100	99	Coastal sand under *Ammophila arenaria*, France	Isolation of Acremine and Heptapeptides
MEF021 (KT315411)	*Torula herbarum* CBS 246.57 (KR873260)	99	**88**	*Brassica oleracea* var. *capita*-*purpurea*, Wageningen, Netherlands	
MEF022 (KT315412)	*Acremonium persicinum* JCM 23083 (NR_131260)	98	99	Coastal sand under *Ammophila arenaria*, France	Isolation of Acremine and Heptapeptides
MEF040 (KT315413)	*Cladosporium funiculosum* CBS 122129 (NR_119845)	100	100	Leaf of *Vigna umbellata*, Japan	
MEF041 (KT315414)	*Cladosporium oxysporum* CBS 125991 (HM148118)	100	98	Soil, terracotta gravene, China	Bioremediation of natural oil spills or other contaminants in tropical environments
MEF043 (KT315415)	*Chordomyces antarcticum* M27 (KJ443241)	93	99	Alkaline soil near Karakul lake, Russia	NA
MEF044 (KT315416)	*Chordomyces antarcticum* M27 (KJ443241)	79	**92**	Alkaline soil near Karakul lake, Russia	NA
MEF045 (KT315417)	*Aspergillus niger* ATCC 16888 (AY373852)	100	100	NA	NA
MEF046 (KT315418)	*Trichoderma neokoningii* GJS 04-216 (DQ841734)	100	99	*Alnus glutinosa*, rotting wood, Peru	NA
MEF047 (KT315419)	*Coprinopsis calospora* CBS 612.91 (GQ249275)	97	99	Soil under *Yucca* sp. in flowering pot, Leiden, Rijksherbarium, Netherlands	NA
MEF048 (KT315420)	*Cladosporium funiculosum* CBS 122129 (NR_119845)	100	100	Leaf of *Vigna umbellata*, Japan	NA
MEF050 (KT315421)	*Cladosporium colocasiae* ATCC 200944 (AF393694.2)	98	98	Necrotic needles of *Pinus ponderosa* trees in Patagonia, Argentina	NA
MEF051 (KT315422)	*Penicillium citrinum* NRRL 1841 (NR_121224)	100	100	NA	NA
MEF053 (KT315424)	*Aspergillus jensenii* NRRL 58600 (JQ301892)	99	99	NA	Calmodulin
MEF054 (KT315425)	*Aspergillus sydowii* CBS 593.65 (NR_131259)	99	99	Human lesion, New York, USA	Causes invasive pulmonary aspergillosis, producer of hepatotoxic and carcinogenic mycotoxin sterigmatocystin, xylanase, xanthones, fellutamides, and anthraquinone
MEF055 (KT315426)	*Aspergillus sydowii* CBS 593.65 (NR_131259)	98	99	Chestnut seed	Sterigmatocystin
MEF056 (KT315427)	*Cladosporium varians* CBS126362 (NR_119856)	100	99	Leaves of *Catalpa bungei*, St. Petersburg, botanical garden of the academy, Russia	NA
MEF058 (KT315428)	*Cladosporium phaenocomae* CBS 128769 (NR_119950)	100	100	Flower of *Phaenocoma prolifera*, Western Cape Province, South Africa	NA
MEF059 (KT315429)	*Plectosphaerella oligotrophica* LC 1990 (JX508810)	100	**95**	*Alisma plantago-aquatica* (Plant)	NA
MEF061 (KT315430)	*Chordomyces antarcticum* M27 (KJ443241)	92	99	Alkaline soil near Karakul lake, Russia	NA
MEF063 (KT315432)	*Aspergillus jensenii* NRRL 58600 (JQ301892)	99	98	Human lesion, New York, USA	Causes invasive pulmonary aspergillosis, producer of hepatotoxic and carcinogenic mycotoxin sterigmatocystin, xylanase, xanthones, fellutamides, and anthraquinone
MEF064 (KT315433)	*Preussia persica* CBS 117680 (GQ292750)	93	**97**	Dead barley leaf, East Azerbaijan, Sarab, Iran	NA
MEF066 (KT315434)	*Aspergillus sydowii* CBS 593.65 (NR131259)	96	99		Calmodulin
MEF067 (KT315435)	*Chordomyces antarcticum* M27 (KJ443241)	94	99	*Hypogymnia physodes* and *Hobsonia christiansenii*, Luxembourg	NA
MEF068 (KT315436)	*Cladosporium colocasiae* ATCC 200944 (AF393694.2)	99	99	Necrotic needles of *Pinus ponderosa* trees in Patagonia, Argentina	NA
MEF069 (KT315437)	*Chordomyces antarcticum* M27 (KJ443241)	95	99	Alkaline soil near Karakul lake, Russia	NA
MEF070 (KT315438)	*Sarocladium strictum* CBS 346.70 (GQ376096.2)	97	99	Old leaf, infested with *Puccinia* sp., *Triticum aestivum*, Schleswig-Holstein, Kiel-Kitzeberg, Germany	NA
MEF071 (KT315439)	*Aspergillus sydowii* CBS 593.65 (NR_131259)	93	99	NA	Several human diseases, including aspergillosis, onychomycosis, and keratomycosis. Several indole alkaloids, Cyclotryprostatin E
MEF073 (KT315440)	*Cladosporium halotolerans* CBS 119416 (NR_119605)	95	99	Hyper saline water of salterns, Namibia	Also isolated from bathrooms and dolphin skin
MEF078 (KT315441)	*Curvularia nicotiae* CBS 655.74 (KJ909772)	90	**95**	Desert soil, Tassili, Algeria	Phytopathogens as well as opportunistic pathogens on human and animals
MEF079 (KT315442)	*Cladosporium phaenocomae* CBS 128769 (NR_119950)	100	100	Flower of *Phaenocoma prolifera*, Western Cape Province, Hermanus, Fernkloof Nature Reserve South Africa	NA
MEF082 (KT315443)	*Trichoderma erinaceum* ATCC MYA-4844 (NR_111837)	100	99	*Alnus glutinosa*, rotting wood, Peru	NA
MEF091 (KT315444)	*Cladosporium phaenocomae* CBS 128769 (NR_119950)	99	99	Flower of *Phaenocoma prolifera*, Western Cape Province, Hermanus, Fernkloof Nature Reserve South Africa	NA
MEF095 (KT315445)	*Aspergillus niger* ATCC 16888 (AY373852)	100	100		NA
MEF101 (KT315446)	*Cladorrhinum microsclerotigenum* CBS 290.75 (FN662475)	99	**93**	Given acc no represents *Cladorrhinum phialophoroides* in CBS database, and *C. microsclerotigenum* is not published, Adana, Turkey	
MEF102 (KT315447)	*Cladorrhinum microsclerotigenum* CBS 290.75 (FN662475)	96	**93**	NA	NA
MEF103 (KT315448)	*Cladorrhinum microsclerotigenum* CBS 290.75 (FN662475)	96	**93**	NA	NA
MEF104 (KT315449)	*Cladorrhinum microsclerotigenum* CBS 290.75 (FN662475)	96	**93**	NA	NA
MEF105 (KT315450)	*Aspergillus sydowii* CBS 593.65 (NR_131259)	97	99	NA	several human diseases, including aspergillosis, onychomycosis, and keratomycosis. Several indole alkaloids, Cyclotryprostatin E
MEF106 (KT315451)	*Fusarium equiseti* NRRL 26419 (NR_121457)	100	99	Soil, Braunschweig, Germany	Mycotoxin production,
MEF109 (KT315453)	*Cladorrhinum microsclerotigenum* CBS 290.75 (FN662475)	96	**92**	NA	
MEF110 (KT315454)	*Fusarium equiseti* NRRL 26419 (NR_121457)	99	98	Soil, Braunschweig, Germany	Mycotoxin production
MEF111 (KT315455)	*Cladosporium halotolerans* CBS 119416 (NR_119605)	95	99	Hyper saline water of salterns, Namibia	Also isolated from bathrooms and dolphin skin
MEF112 (KT315456)	*Fusarium nygamai* NRRL 13448 (NR_130698)	100	99	Necrotic root, *Sorghum bicolor*, Narrabri, New South Wales	Lymphoblastic non Hodgkin's lymphoma, bioherbicide
MEF113 (KT315457)	*Fusarium equiseti* NRRL 26419 (NR_121457)	100	99	*Cynodon lemfuensis*	Spikelet disease to plant
MEF115 (KT315458)	*Fusarium equiseti* NRRL 26419 (NR_121457)	100	99	Soil, Braunschweig, Germany	Mycotoxin production
MEF116 (KT315459)	*Cladorrhinum microsclerotigenum* CBS 290.75 (FN662475)	99	**93**	NA	NA
MEF117 (KT315460)	*Aspergillus niger* ATCC 16888 (AY373852)	100	100	NA	NA
MEF118 (KT315461)	*Microdiplodia hawaiiensis* CBS 120025 (DQ885897)	99	**95**	Stem, *Sophora chrysophylla*, Saddle Road, Hawaii	NA
MEF119 (KT315462)	*Penicillium biforme* CBS 297.48 (KC411731)	100	100	Cheese, Connecticut, Stovis, USA	NA
MEF121 (KT315463)	*Cladorrhinum microsclerotigenum* CBS 290.75 (FN662475)	100	**92**	NA	NA
MEF122 (KT315464)	*Pseudopestalotiopsis cocos* CBS 272.29 (KM199378)	100	**86**	*Cocos nucifera*, Buitenzorg, Java	NA
MEF123 (KT315465)	*Acremonium furcatum* CBS 122.42 (AY378154)	99	**95**	Dune sand under *Convolvulus soldanella*, Normandie, Pointe du Siège, France	Antimicrobial metabolites
MEF124 (KT315466)	*Coprinopsis calospora* CBS 612.91 (GQ249275)	100	99	Soil under *Yucca* sp. in flowering pot, Leiden, Rijksherbarium, Netherlands	NA
MEF125 (KT315467)	*Aspergillus sydowii* CBS 593.65 (NR_131259)	97	99	NA	Several human diseases, including aspergillosis, onychomycosis, and keratomycosis. Several indole alkaloids, Cyclotryprostatin E
MEF126 (KT315468)	*Fusarium equiseti* NRRL 26419 (NR_121457)	100	99	*Cynodon lemfuensis*	Spikelet disease to plant
MEF127 (KT315469)	*Cladorrhinum microsclerotigenum* CBS 290.75 (FN662475)	100	**92**		NA
MEF128 (KT315470)	*Fusarium equiseti* NRRL 26419 (NR_121457)	100	99	*Cynodon lemfuensis*	Spikelet disease to plant
MEF129 (KT315471)	*Cladorrhinum microsclerotigenum* CBS 290.75 (FN662475)	100	**92**		NA
MEF130 (KT315472)	*Penicillium chrysogenum* ATCC 10106 (HQ026745)	100	99	Cheese, Connecticut	Also found on salted food products, produces penicillin and xanthocillin X
MEF131 (KT315473)	*Penicillium chrysogenum* ATCC 10106 (HQ026745)	100	100	Cheese, Connecticut	Also found on salted food products, produces penicillin and xanthocillin X
MEF132 (KT315474)	*Penicillium chrysogenum* ATCC 10106 (HQ026745)	100	100	Cheese, Connecticut	Also found on salted food products, produces penicillin and xanthocillin X
MEF133 (KT315475)	*Cladorrhinum microsclerotigenum* CBS 290.75 (FN662475)	100	**93**	NA	NA
MEF134 (KT315476)	*Cladorrhinum microsclerotigenum* CBS 290.75 (FN662475)	97	**93**	NA	NA
MEF135 (KT315477)	*Cladosporium oxysporum* CBS 125991 (HM148118)	100	99	Soil, terracotta gravene, China	Bioremediation of natural oil spills or other contaminants
MEF136 (KT315478)	*Cladosporium oxysporum* CBS 125991 (HM148118)	100	99	Soil, terracotta gravene, China	Bioremediation of natural oil spills or other contaminants
MEF137 (KT315479)	*Aspergillus niger* ATCC 16888 (AY373852)	100	100	NA	NA
MEF138 (KT315480)	*Cladorrhinum microsclerotigenum* CBS 290.75 (FN662475)	100	**93**	NA	NA
MEF140 (KT315481)	*Curvularia heteropogonis* CBS 284.91 (HF934919)	100	**96**	Hyper saline water of salterns, Namibia	Also isolated from bathrooms and dolphin skin
MEF141 (KT315482)	*Cladorrhinum microsclerotigenum* CBS 290.75 (FN662475)	100	**92**	NA	NA
MEF142 (KT315483)	*Cladorrhinum microsclerotigenum* CBS 290.75 (FN662475)	97	**92**	NA	NA
MEF147 (KT315484)	*Cladosporium halotolerans* CBS 119416 (NR_119605)	91	**96**	Hyper saline water of salterns, Namibia	Also isolated from bathrooms and dolphin skin
MEF148 (KT315485)	*Cladosporium oxysporum* CBS 125991 (HM148118)	100	99	Soil, terracotta gravene, China	Bioremediation of natural oil spills or other contaminants
MEF156 (KT315486)	*Aspergillus sydowii* CBS 593.65 (NR_131259)	100	100	NA	Human diseases like aspergillosis, onychomycosis, and keratomycosis. Produces indole alkaloids, Cyclotryprostatin E
MEF158 (KT315487)	*Fusarium equiseti* NRRL 26419 (NR_121457)	100	99	*Cynodon lemfuensis*	Spikelet disease to plant
MEF159 (KT315488)	*Aspergillus venenatus* NRRL 13147 (JQ301896)	100	99		NA
MEF161 (KT315489)	*Fusarium equiseti* NRRL 26419 (NR_121457)	99	98	Soil, Braunschweig, Germany	Mycotoxin production
MEF166 (KT315490)	*Aspergillus quadrilineatus* NRRL 201 (NR_131289)	97	99	Froidchapelle, Belgium	Lipopeptide antifungal drug, causative agent of aspergillosis in humans and animals
MEF170 (KT315491)	*Cladosporium halotolerans* CBS 119416 (NR_119605)	100	100	Hyper saline water of salterns, Namibia	Also isolated from bathrooms and dolphin skin
MEF174 (KT315492)	*Aspergillus terreus* ATCC 1012 (NR_131276)	98	99	Soil, Connecticut	Produces hydroxylates aniline, antiviral agent, LL-S88 alpha, exo-1,4-beta-D-xylosidase beta-xylosidase, itaconic acid and cis-aconitic acid
MEF176 (KT315493)	*Cladorrhinum microsclerotigenum* CBS 290.75 (FN662475)	100	**93**	NA	NA
MEF177 (KT315494)	*Cladorrhinum microsclerotigenum* CBS 290.75 (FN662475)	100	**93**	NA	NA
MEF178 (KT315495)	*Cladorrhinum microsclerotigenum* CBS 290.75 (FN662475)	98	**92**	NA	NA
MEF179 (KT315496)	*Aspergillus quadrilineatus* NRRL 201 (NR_131289)	96	98	Soil, China	Agent of fungal sinusitis, Onychomycosis
MEF180 (KT315497)	*Fusarium nygamai* NRRL 13448 (NR_130698)	100	99	Necrotic root, *Sorghum bicolor*, Narrabri, New South Wales	Lymphoblastic non Hodgkin's lymphoma, bioherbicide
MEF181 (KT315498)	*Fusarium equiseti* NRRL 26419 (NR_121457)	100	99	Soil, Braunschweig, Germany	Mycotoxin production
MEF190 (KT315499)	*Aspergillus niger* ATCC 16888 (AY373852)	100	100		NA
MEF191 (KT315500)	*Fusarium equiseti* NRRL 26419 (NR_121457)	100	99	Soil, Braunschweig, Germany	Mycotoxin production
MEF194 (KT315501)	*Aspergillus niger* ATCC 16888 (AY373852)	100	100	NA	NA
MEF197 (KT315502)	*Fusarium equiseti* NRRL 26419 (NR_121457)	100	99	Soil, Braunschweig, Germany	Mycotoxin production
MEF201 (KT315503)	*Cladosporium halotolerans* CBS 119416 (NR_119605)	95	99	Hyper saline water of salterns, Namibia	Also isolated from bathrooms and dolphin skin

In preliminary screening, 14 different strains from 4-different genera, and 3 classes showed positive result for phenol oxidase production. Result of comparative study of phenol oxidase production indicated that strain MEF018 showed maximum phenol oxidase secretion at pH 12 whereas strain MEF008, MEF109, and MEF135 showed maximum production at pH 10. The effects of salt, pH, and temperature were also studied on growth of these strains and the data are presented in Figures S2A–C. Based on their enzyme production potential at high salinity and pH, *Fusarium* sp. strain MEF008, *Curvularia* sp. strain MEF018, *Cladorrhinum* sp. strain MEF109, and *Cladosporium* sp. strain MEF135 were selected from the 14 fungal isolates (phenol oxidase producer) for further comparative study of fungal phenol oxidase (Figures S3, S4). Thus, secondary screening was conducted with only 4 strains which were maximum producer in primary screening. Result of enzyme assay indicated that *Curvularia* sp. strain MEF018 produced high levels of phenol oxidase followed by *Cladosporium* sp. strain MEF135, while *Fusarium* sp. strain MEF008 and *Cladorrhinum* sp. strain MEF109 showed nearly five time less phenol oxidase production efficiency than above two strains (Figures S5, S6). In addition *Curvularia* sp. strain MEF018 showed optimum phenol oxidase production potential at high pH (pH 12) and at 40°C, which is good for ecological point of view and showed that these potential of strains could be exploited in extreme condition of alkalinity, salinity and temperature (Figures S5, S6). Analysis of data indicated that strain MEF018 and strain MEF109 showed similar amount of cellular biomass at third day but showed big difference in phenol oxidase production while strain MEF135 showed lower cellular biomass than strain MEF109 but produced four to five time more enzymes. Thus, data indicated that quantity of extracellular enzyme production is not related to cellular biomass but it depends on enzyme production potential of the cells. We found that, in addition to efficient phenol oxidase production potential in extreme condition of alkalinity (pH- 12) and at high temperature (40°C) *Curvularia* sp. strain MEF018 showed low sequence similarity (96%) with previously isolated and characterized *Curvularia pseudorobusta*. It indicated that strain MEF018 is a novel extremophilic species of genus *Curvularia* with immense ecological and biotechnological importance and selected for further in-depth taxonomical characterization.

**Figure 3:**
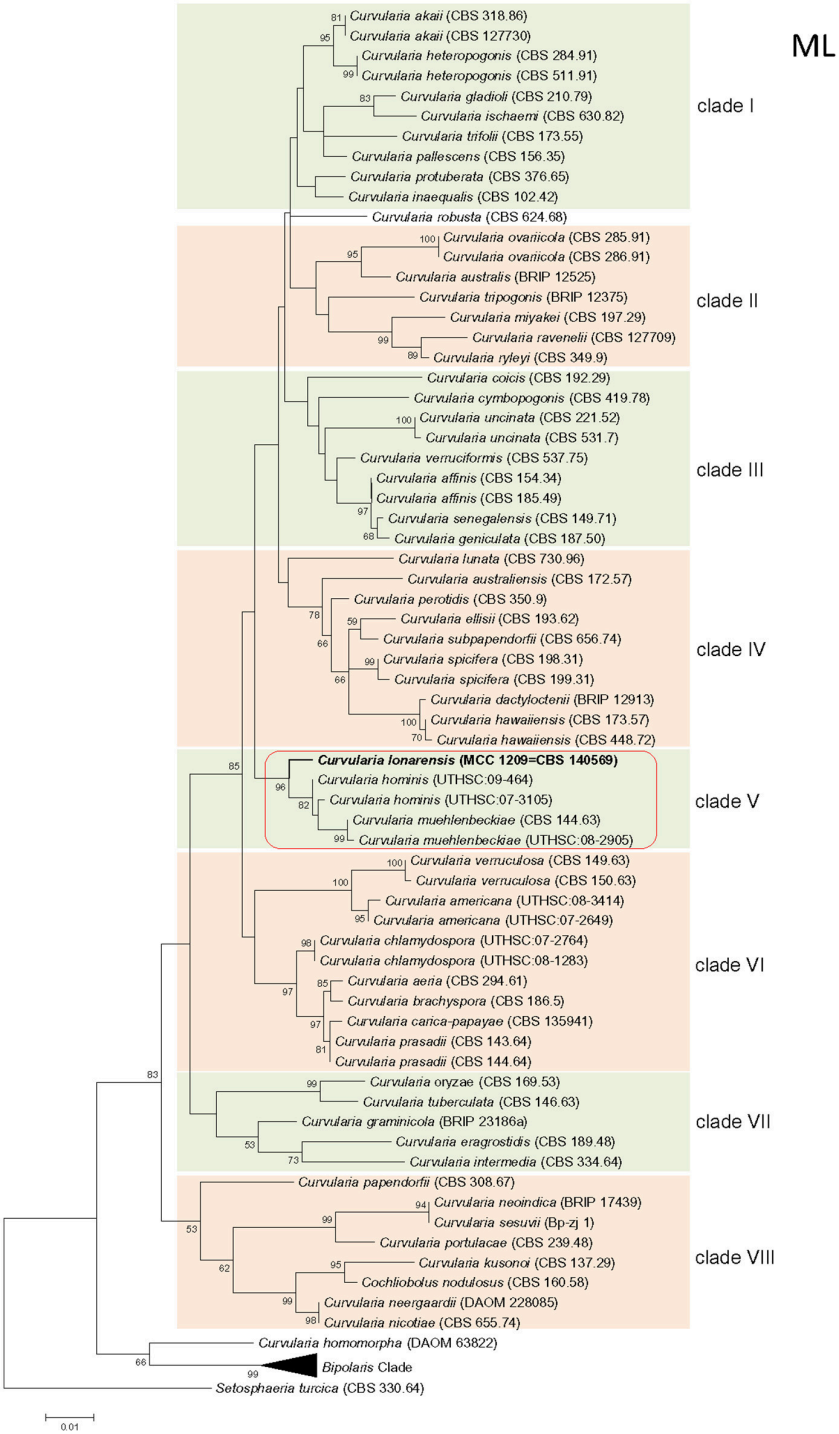
**Maximum Likelihood (ML) tree of the ***Curvularia*** strains studied**. Bootstrap support of branches indicated on the node was obtained using 1000 replicates. Only statistically significant bootstrap values (≥50%) are indicated. Branch lengths are indicated as 0.01 substitutions per positions according to the scale bar underneath the tree. Number on right side of species name denotes the strain number/ culture collection accession number. Number in parentheses denotes accession number of the sequence deposited to online database.

Phylogenetic tree constructed using concatenated alignment of ITS-LSU-*gpd* sequences from 81 strains of *Curvularia* and *Bipolaris* derived from Madrid et al. (2014) and Manamgoda et al. (2014) along with strain MEF018^T^ from this study showed that it clustered with two species of *Curvularia, C. hominis* and *C. muehlenbeckiae* (Figure 3). Although the ITS sequence of strain MEF018^T^ showed highest sequence similarity with *C. pseudorobusta* (96%), the *gpd* gene sequence showed highest sequence similarity with *C. perotidis* and *C. neerdaardii* (93%) and D1/D2 region of LSU showed no differentiation (99% similarity with most species), it formed sister clade with *C. muehlenbeckiae* CBS 144.63^T^ and *C. hominis* UTHSC 09-464^T^. All three species together made separate clade in ML based phylogenetic tree. Even, use of other two methods (NJ and MP) also gave similar tree topologies (tree not included). Overall, the tree showed 8-clades. The strain *C. lonarensis* MEF018^T^ belongs to clade-V along with *C. hominis* and *C. muehlenbeckaie*. The results of phylogenetic analyses of ITS, LSU and *gpd* gene separately as well as combined dataset along with number of bases, number of parsimony informative characters and other parameters for best substitution model is compiled and presented in Table 3. Thus, the result of phylogenetic study indicated that the strain MEF018 is distantly related with previously cultivated members of the genus *Curvularia* and is a novel species (Figure 3). Details of morphological, physiological and taxonomic features of the novel fungal species are discussed below.

**Table 3:** **Statistics resulting from phylogenetic analyses of ITS, LSU, and ***gpd*****.

**Dataset**	**No. of taxa**	**Number of characters^b^ included in analysis (including gaps)**	**ML**			
			**Number of parsimony informative characters^b^ (PIC) (%)**	**Number of conserved characters^b^ (C)**	**Number of variable characters^b^ (V)**	**Number of Singleton (S)**
LSU^a^	74	852	28	808	44	16
ITS^a^	82	783	197	477	284	85
*gpd*^a^	79	564	212	327	237	25
LSU-ITS-*gpd*^c^	82	2203	437	1612	565	126

a *ITS, internal transcribed spacers and intervening 5.8 S nrDNA; LSU, large subunit; gpd, partial glyceraldehyde-3-phosphate dehydrogenase gene*.

b *characters including base pairs and gaps*.

c *Nucleotide substitution mode- Kimura 2-parameter model; Statistical Method- Maximum Likelihood; Phylogeny Test- Bootstrap method*.

### Description of *Curvularia lonarensis* Rohit Sharma and Rahul Sharma sp. nov.

**Figure 4:**
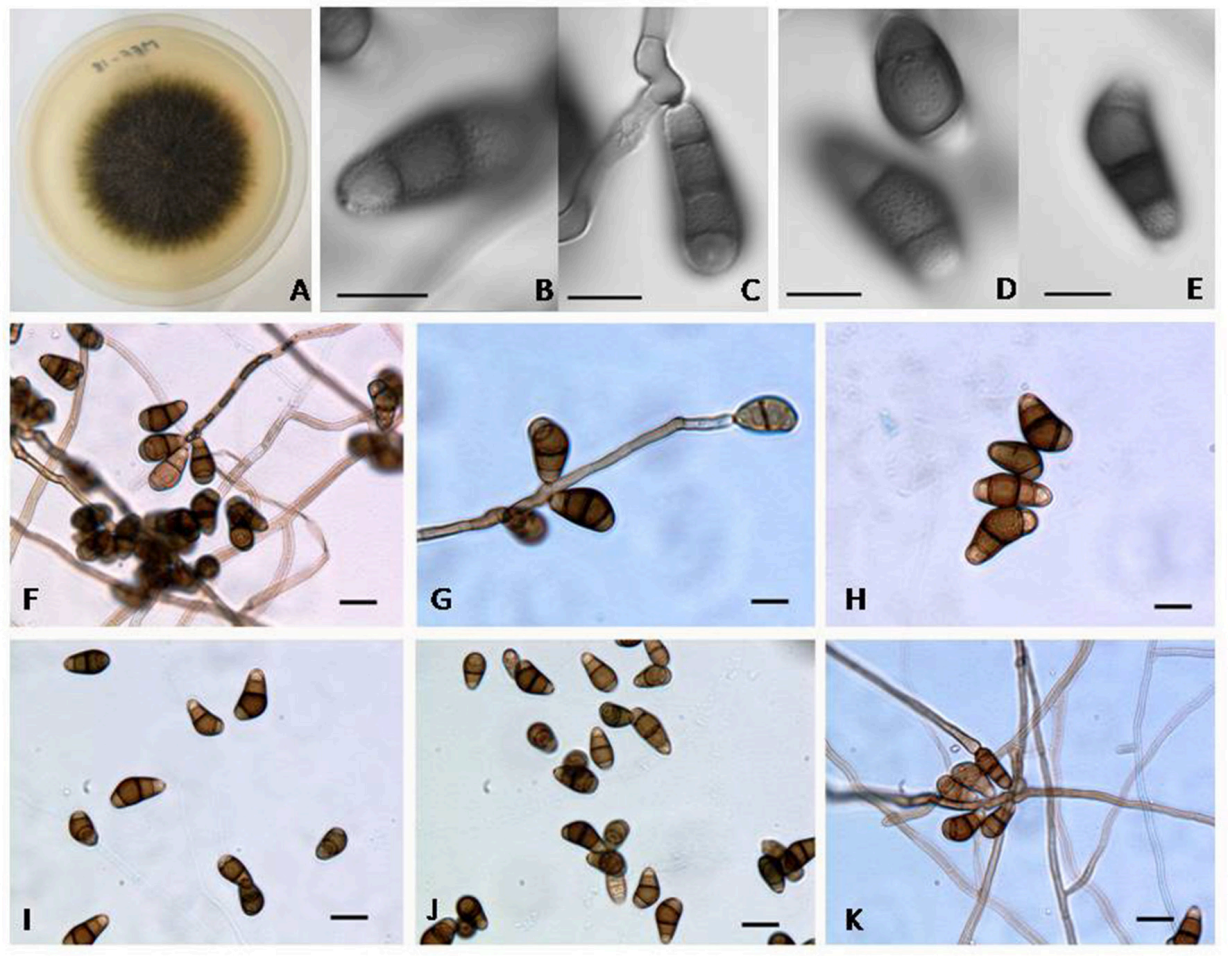
**(A)** Colony morphology of *Curvularia lonarensis* strain MEF018 on OA; **(B–E)** Morphology of conidia as captured by DIC imaging showing ornamentation on all cells of conidia (Scale bars: **B–E** = 10 μm); **(F–K)** Structural features of conidia and conidiophores of *Curvularia lonarensis* strain MEF018 as depicted during light microscopic imaging. (scale bars: **F–K** = 20 μm).

*Curvularia lonarensis* Rohit Sharma and Rahul Sharma **sp. nov**. (Figure 4).

MycoBank: MB 814557.

#### Etymology

The epithet *lonarensis* is derived from the Lonar lake situated in Buldhana district of Maharashtra, India from where the fungus was isolated.

#### Vegetative hyphae

Vegetative hyphae are septate, branched, hyaline to sub-hyaline, smooth-walled and ranges from 3.2 to 4.9 μm (width). *Conidiophores* are septate, simple to branched, semi–macronematous, mononematous, straight, or flexuous, geniculate toward the apex, subhyaline to dark brown, smooth to aperculate with cell wall often thicker than those of the vegetative hyphae. Size of *Conidiophores* ranged from 40–306.5 μm (length) × 4.4–6.2 μm (width), with subnodulose and nodulose intercalary swellings ranging from 3.9 to 6.0 μm (width) which coincide with conidiogenous cells. Conidiogenous cells are subcylindrical to irregularly shaped and integrated with terminal and intercalary conidiophores cells. They are monopolytrectic, proliferating sympodially, and size of intercalary conidiogenous cells are ranging from 4.6–21.0 μm (length) × 3.4–5.7 μm (width) whereas the size of terminal conidiogenous cells range 8.3–14.7 μm (length). *Conidia* of strain MEF018 are 4–celled, asymmetrical to curve and their size ranged from 19.2–30.7 μm (length) × 10.7–15.0 μm (width). Middle cells are dark brown and usually verruculose while the cells located at terminal ends are paler, and ornamented. *Chlamydospores and microconidiation* were not observed.

#### Cultural characteristics

It forms 66 and 77 mm wide colonies on MEA and OA respectively after 5 days of incubation at 30°C. Colonies are flat, circular, filamentous, greenish-black on OA but grayish-green on MEA with pale colored margin and lavish sporulation. Reverse side of the colonies showed blackish pigmentation with pale colored margin. Colonies on CDA and PDA are 68 and 71 mm wide respectively after 5 days at 30°C. It forms grayish-green colonies on CDA and greenish-black on PDA. Colonies on both the media looks circular, filamentous, flat, with slightly hairy mycelia, spreading, white margins, and with full of sporulation. Reverse view of the colonies have grayish-black spots. Colonies on SDA are 73 mm wide after 5 days of incubation at 30°C, looks grayish-green slightly raised, with pale colored margins, and with abundant aerial mycelia.

*Sexual morph:* Not observed.
*Habitat:* Hyper alkaline and saline Lonar lake.
*Distribution:* Buldhana (Maharashtra, India).
*Type:* INDIA, Maharashtra, Buldhana, Lonar, from water and sediment of Lonar lake, 01 Oct. 2010, Rohit Sharma (**holotype** CBS 140569^T^ = MCC 1209^T^ = MEF018^T^).
*Gene sequences ex-holotype:* KT315408 (ITS); KY007019 (*gpd*); KY007018 (LSU).

The genus *Curvularia* was first time described by Schmidt and Kunze (1817) with *Curvularia lunata* as type species of the genus. It is characterized by production of transversely septate conidia with dark hila, which is asymmetrically curved from middle cell. Its closest genus is *Bipolaris* which forms symmetrically swollen central cell (distoseptate) while *sensu stricto* species of *Curvularia* lack this feature. Traditionally, both the genera (*Bipolaris* and *Curvularia*) were distinguished by conidial features but molecular data have now confirmed their positions in the family *Pleosporaceae* order *Pleosporales*, (Zhang et al., 2009, 2012). Manamgoda et al. (2012) reclassified several *Bipolaris* and *Curvularia* species based on phylogeny of ITS, LSU, *gpd, tef* sequences. Following re-classification, some of the plant pathogenic species of *Bipolaris* were shifted to *Curvularia* (da Cunha et al., 2013; Madrid et al., 2014). Even the genus *Pseudocochliobolus* was merged in *Curvularia* with type species *P. nisikadoi* now described as *Curvularia coicis*. Manamgoda et al. (2014) revised the generic boundaries between *Bipolaris* and *Curvularia* based on ITS and *gpd* phylogenetic tree. Similar to the present study, Manamgoda et al. (2012); Manamgoda et al., 2014 found two major groups, one group includes species of *Bipolaris* and other group include species of *Curvularia* (having 8 clades). Thus, the phylogenetic analysis (Figure 3) clearly shows that the sequences of ITS-LSU-*gpd* combined dataset resolves the two genera as well as species within individual genus. Although single name of both morphs have reduced the complexities in fungal taxonomy of many genera, lack of authentic, curated database of sequences had made it difficult for correct identification of species (Sharma, 2012).

According to our observation the strain MEF018 belong to the genus *Curvularia* because it forms curved conidia with dark enlarged, central cell and double layered wall. As per Manamgoda et al. (2012), conidia of *Curvularia* can be straight or curved. When curved, the conidia have enormously enlarged intermediate cells contributing to their curvature. Similar to *C. americana, C. tuberculata* and *C. verruculosa* the surface of the conidia of the strain MEF018 is rough (Figures 4B–E). The conidia of the strain MEF018 is slightly larger (19–30 × 10–15 μm) than the conidia of *C. americana* (13–28 × 7–15 μm) but smaller than *C. tuberculata* (23–52 × 13–20 μm) and *C. verruculosa* (20–40 × 12–17 μm). Differential features of MEF018 with phylogenetically closest relatives of the genus *Curvularia* are given in the Table 4.

**Table 4:** **Comparison of morphological characters of ***Curvularia lonarensis, C. hominis, C. muehlenbeckiae*****.

**Character**	***C. lonarensis* (Present study)**	***C. hominis* (Madrid et al., 2014)**	***C. muehlenbeckiae* (Madrid et al., 2014)**
Colony morphology	Centre- dark green- black	Centre- dark green	Centre- pale gray
	Margin- pale colored,	Margin- olive to white	Margin- dark olive
	Reverse- blackish	Reverse- olive-dark green	Reverse- olivaceous-black
Vegetative hyphae size	Smooth-walled, 4 μm (3.2– 4.9 μm) wide	Smooth- slightly asperulate, 1.5–5 μm wide	Smooth-walled, 1.5–5 μm wide
Conidial Structure	No. of Cell- 4	No. of Cell- 4–5	No. of Cell- 4
	Size- 25 μm (19–30) × 13 μm (11–15)	Size- 18–30 × 7–14 μm	Size- 17–26 × 8.5–12 μm
	Intermediate cells- usually finely verruculose and dark brown	Intermediate cells- usually verruculose and darker, brown	Intermediate cells- usually verruculose, dark brown
	End Cells- paler and ornamented	End Cells- subhyaline- pale brown, smooth-walled	End Cells- paler and smooth-walled or less ornamented
Structure of conidiophores	Size- 167.4 μm (40– 306.5) × 5 μm (4.4– 6.2)	Size 55–325 × 2–5 μm	Size- 21.5–398 × 2–5 μm
	Intercalary swellings- 4.8 μm (3.9–6)	-	Intercalary swellings- 9.5 μm
Conidiogenous cells	Intercalary conidiogenous cells 4.6–21 μm × 3.4–5.7 μm	Intercalary conidiogenous cells 6–26 × 4–9 μm	Intercalary conidiogenous cells 5–18 μm
	Terminal conidiogenous cells 8.3–14.7 μm		Terminal conidiogenous cells 5–25 μm
Temp. optima	30°C	25°C	24°C
			
Sexual morph	Not observed	Not observed	Not observed

Phylogenetic analysis of combined dataset dissects the available species of the genus *Curvularia* in to eight main clades (Figure 3). Study of tree topology indicated that strain MEF018 showed distant relationship with other existing members of the genus *Curvularia* and clustered with *C. hominis* and *C. muehlenbeckiae* with strong bootstrap support (96%). All the three species possess warted or verruculose conidia. The species of *Curvularia* with warted or verruculose conidia appear in different clades outside *C. hominis*-clade (*C. hominis, C. muehlenbeckiae*, strain MEF018), which suggests polyphyletic origin of conidial ornamentation in genus *Curvularia* (Madrid et al., 2014). The *C. hominis* and *C. muehlenbeckiae* species are isolated from human and leaf of *Muehlenbeckia* plant respectively growing optimally at 24°C. Whereas, the strain MEF018 is only isolate among the clade which is isolated from a soda lake and grows at high pH and 30°C temperature. The close clustering of 3 *Curvularia* species belonging to different habitat indicates that ecological gradient may not be a factor in differentiating these species, hence not correlated with fungal diversity. We also observed that the clustering pattern of the phylogenetic tree supports the morphological data of the conidia in the genus *Curvularia*. The clade -IV contains mostly 4-celled, conspicuously distoseptate conidia with darker middle cell. They are mostly curved at the third cell from the base, and larger in size than conidia of the members of the others clade. Morphologically, strain MEF018 is closer to clade IV (consisting of *C. lunata*) as it forms curved conidia with darker third cell. Hence, in the present study, phylogeny of combined dataset along with morphological details gives good resolution to distinguish the strain MEF018 from its close relatives and proves that strain MEF018 is a novel species of the genus *Curvularia*.

Strain MEF018 showed optimum growth on OA among the tested fungal growth media. It tolerated 10% NaCl concentration in the medium but showed optimum growth at 1% (Figure S2a). It showed positive growth between pH 5–14 with optimal mycelial growth at pH- 11 (Figure S7). Evaluation of the pH of the medium after fungal growth indicated that strain MEF018 secreted some metabolites which shift the pH of the growth medium toward its optima (pH 10–11). Temperature range for the growth was between 10 and 40°C with optimum growth at 30°C. Thus, in conclusion based on morphological, physiological and phylogenetic details strain MEF018 sufficiently delineates with existing members of the genus *Curvularia* and proposed as *Curvularia lonarensis* Rohit Sharma & Rahul Sharma sp. nov. In conclusion, the above described novel fungus is an important finding of current study because till date no *Curvularia* species with phenol oxidase producing potential has been reported from hyper alkaline and saline habitats.

## Discussion

**Figure 5:**
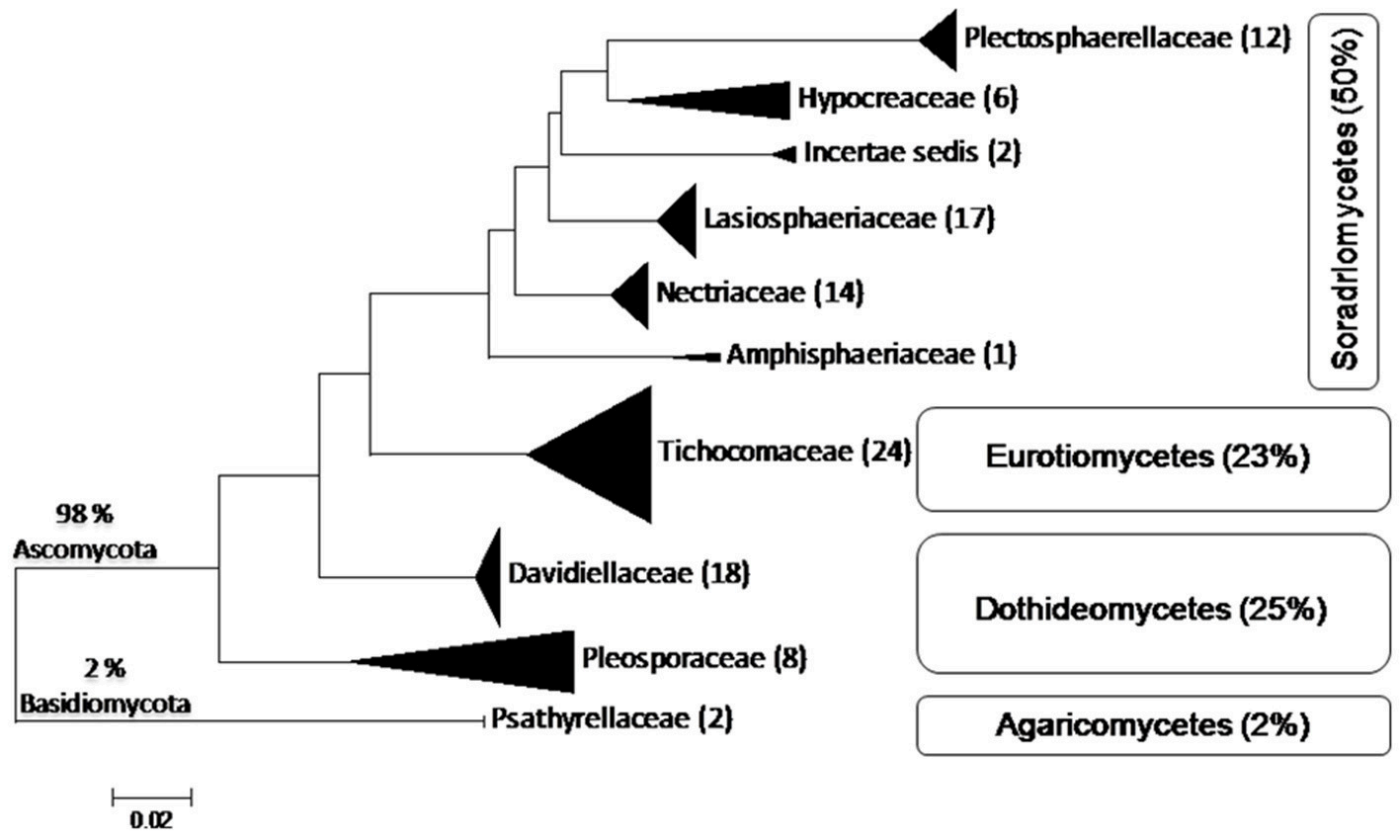
**Schematic representation of placement of 104 isolates belonging to 38 species derived from ITS sequences of fungi from Lonar lake, Buldhana, Maharashtra, India**. Classification follows Hibbett et al. (2007). All isolates belong to *Ascomycota* except 2% belonging to *Basidiomycota*. The isolates of *Ascomycota* are distributed among three classes, *Sordariomycetes, Dothideomycetes*, and *Eurotiomycetes*. Percentages indicate the total number of isolates out of the 104 isolated fungi.

Lonar lake is a soda lake located in Buldhana district of Maharashtra, India but understudied in terms of fungal diversity. Available literature indicated that except a single report on keratinophillic fungi from the soil of slope by Deshmukh and Verekar (2006) very little or no information on the soda lake fungi from Lonar lake are available. Generally, fungi prefer acidic, or neutral pH, and very few data are available on extremophilic fungi especially from soda lakes. Therefore, isolation of alkaliphilic fungi from Lonar lake and other such habitats would be beneficial to improve the database as well as for their future commercial exploitation. Due to commercial and physiological importance of extremophiles fungi several other groups are working on this aspect from various soda lakes across the world (Table 5). Our study also demonstrated that a wide range of fungal diversity inhabit in the hyper alkaline and saline habitat of Lonar lake. Furthermore, all the fungal isolates recovered from Lonar lake are members of the Dikarya, most of them (98%) belong to *Ascomycota*, and are distributed throughout the sub-phylum *Pezizomycotina* (Figure 5). Phylogenetic analyses reveals that alkaliphilic trait is widely distributed among the various sub-phyla of *Ascomycota* suggesting that diverse groups of fungi have the potential to adopt themselves in extremophilic conditions of the various soda lakes. While, phylum *Basidiomycota* was represented by a single member *Coprinopsis* sp. (2 strains) belonging to family *Psathyrellaceae*. The species of *Coprinopsis* generally inhabits terrestrial habitat growing on either coprophilous or lignicolous substrate. Strains MEF047 and MEF124 showed closest similarity with *Coprinopsis calospora* (≡ *Coprinopsis calosporus*) which was isolated from a stem in flowerpot from Netherlands. In Lonar lake it is possible that they were associated with some dead wood debris inside the lake. Sequence similarity data from present study indicated that most of the strains isolated showed similarity with previously characterized strains isolated from alkaline, saline, or other extreme environments (Table 2) and confirm that the isolated strains are native to the lake samples and not contaminant of isolation procedures. In addition, out of 38 species reported from current study, 12 are putative novel (involving 32 strains) based on the current criteria (≤97% sequence similarity with closest relative) set for fungal species delineation by Blaalid et al. (2013), which suggest that Lonar lake is an important reservoir for the ecologically and economically important fungi and need further investigation in terms of physiology and genetics to explore their role in biogeochemical cycling and to get the valuable products of industrial importance.

**Table 5:** **Comparison of present and previous fungal studies on Soda lake around the world**.

**Kladwang et al. (2003)**	**Grum-Grzhimaylo et al. (2016)**	**Present study (2016)**
*Acremonium*	*Acremonium*	*Acremonium*
*Fusarium*	*Fusarium*	*Fusarium*
–	*Alternaria*	*Alternaria*
–	*Chordomyces*	*Chordomyces*
–	*Cladosporium*	*Cladosporium*
–	*Penicillium*	*Penicillium*
*Verticillium*	*Verticillium*	–
–	*Sarocladium*	*Sarocladium*
–	*Emericellopsis*	***Aspergillus***
*Gliomastix*	*Acrostalagmus*	***Coprinopsis***
*Metarrhizium*	*Lasiosphaeriaceae* sp.	***Curvularia***
*Mucor*	*Pleosporaceae* sp.	***Cladorrhinum***
*Paecilomyces*	*Purpureocillium*	***Microdiplodia***
*Phialophora*	*Scopulariopsis*	***Plectosphaerella***
*Scopulariopsis*	*Sodiomyces*	***Preussia***
*Stilbella*	*Thielavia*	***Pseudopestalotiopsis***
–	–	***Torula***
–	–	***Trichoderma***
Total strains: 490	Total strains: 100	Total strains: 104

*Genera mentioned in the bold face are first time reported from hyper alkaline and saline habitat during this study*.

The Table 6 shows diversity of fungal species isolated from various hyper alkaline and hyper saline environments across the world along with data on the habitats of isolation. Among the isolated strains in present study, members of *Acremonium, Alternaria, Chordomyces, Cladosporium, Fusarium, Penicillium* have also been isolated from other soda lakes supported the findings of our study. In addition a total of 10 genera including *Aspergillus, Cladorrhinum, Coprinopsis, Curvularia, Microdiplodia, Plectosphaerella, Preussia, Pseudopestalotiopsis, Torula*, and *Trichoderma* were unique to this study and reported from Lonar lake only (Table 5). We also observed that *Cladorrhinum* with 17 different isolates dominate among the isolated strain which is not reported from any soda lake in earlier studies (Tables 5, 6). Furthermore, studies conducted on soda lakes from other part of the world reported lesser diversity than reported in current study (Kladwang et al., 2003; Grum-Grzhimaylo et al., 2016). Thus, our diversity data indicated that Lonar lake harbor wide range of unique fungal diversity and also indicated that these strains are indigenous to alkaline habitat of Lonar lake.

**Table 6:** **Fungal species isolated from various hyper alkaline and hyper saline environments around world with the data on the habitats they were isolated from**.

**Taxon**	**Habitat and isolation place**	**pH**
*Acremonium alcalophilum*^♢^	Manure	alkaliphilic
*A. roseolum*^#^	Kuchiger area, Trans-Baikal, Russia	9
*A. rutilum*^†^	Saline soil, Lake Baikal basin, Russia	alkaliphilic
*A. sclerotigenum*^#^	Near Alla River, Trans-Baikal, Russia	8
*Acremonium* sp.^¶^^#^	Alkaline limestone caves, Japan; Grassland, Indonesia; Orongoyskoe Lake, Kulunda Steppe, Altai, Russia; Aral Lake, Kazakhstan/ Uzbekistan	7.8–10.3
Acrostalagmusluteo albus^*^	Lake of Solyonoe, Zheltir, Bezimyannoe, Glauberovoe, Kulunda Steppe, Russia	9.5–10
Alternaria alternata^*^	Petuchovskoe Lake, Kulunda Steppe, Altai, Russia	10.1
A. kulundii^*^	Tanatar Lake and Uzkoe Lake, Kulunda Steppe, Altai, Russia	9.8–10.2
A. molesta^*^	Tanatar Lake, Kulunda Steppe, Altai, Russia	
A. petuchovskii^*^	Petuchovskoe Lake, Kulunda Steppe, Altai, Russia	9.9
A. shukurtuzii^*^	Shukurtuz Lake, Kulunda Steppe, Altai, Russia	9.9
*Alternaria* sp.^*^	Solyonoe Lake, Kulunda Steppe, Altai, Russia	10
*Aspergillus oryzae*^◙^	Soil samples	9–9.5
Chordomyces antarcticum^*^	Lake of Uzkoe, Solyonoe, Bezimyannoe, Karakul, Petuchovskoe, Berdabay, Kulunda Steppe, Altai, Russia; Nuhe-Nur Lake, Trans-Baikal, Russia; Bayan-Zag area, North Gobi, Mongolia; Aral Lake, Kazakhstan	8.9–10.1
Cladosporium sphaerospermum^*^	Near Alla River, Trans-Baikal, Russia	8
*Emericellopsis alkalina*^#^	Lake of Berdabay, Bezimyannoe, Mirabilit, Noname (near Sazadi Lake), Solyonoe, Shukurtuz, Tanatar, Zheltir, Kulunda Steppe, Altai, Russia; Nuhe-Nur Lake and Sulfatnoe Lake, Trans-Baikal, Russia; Choibalsan, North-East Mongolia	9.5–11
*E. maritima*^#^	Sea water	–
*E. minima*^#^	Mangrove water	–
*E. pallida*^#^	Sea water	8.3
*Exophiala alkalophila*^$^	Soil samples	10.4
*Fusarium bullatum*^†^	Soil	11
*F. oxysporum*^‡^	Soil	11
*F. solani*	Soda lake	
*Fusarium* sp.^¶^^*^	Aral Lake, Kazakhstan; Alkaline limestone caves, Japan; Grassland, Indonesia	8
*Gliocladium* sp.^¶^	Alkaline limestone caves, Japan; Grassland, Indonesia	alkaline medium
*Lasiosphaeriaceae* sp.^*^	Choibalsan area, North-East Mongolia Burd Lake	10.1
*Penicillium variables*		11
*Penicillium* sp.^*^	Trans-Baikal, Russia Sulfatnoe Lake	8.1
*Plectosporium* sp.^¶^	Alkaline limestone caves, Japan; Grassland, Indonesia	alkaline medium
*Pleosporaceae* sp.^*^	Petuchovskoe Lake, Belen'koe Lake, noname lake and Tanatar Lake Kulunda Steppe, Altai, Russia; Ulan-Nur Lake, North Gobi, Mongolia	7.8–10.1
Purpureocillium lilacinum^*^	Nuhe-Nur Lake, Trans-Baikal, Russia	10.1
*Sarocladium* sp.^#^	Aral Lake, Kazakhstan Aktumsyk Cape	8.3
Scopulariopsis brevicaulis^*^	Mirabilit Lake, Kulunda Steppe, Altai, Russia; Kuchiger, Trans-Baikal, Russia	8–9.7
S. fusca^*^	Trans-Baikal, Russia Kuchiger	9
*Sodiomyces alkalinus*^#^	Lake of Bezimyannoe, Tanatar, Karagay, Gorkoye, Petuchovskoe, Solyonoe, Karakul, Mirabilit, Kulunda Steppe, Altai, Russia; Low-salt soda lake, Steppe, Chitinskaya area, Russia; Soda soil, Natron Lake, Tanzania; Barun-Undziyn Lake, North-East Mongolia; Bayan-Zag area, North Gobi, Mongolia; Shar-Burdiyn Lake, Choibalsan area, North-East Mongolia	9.2–10.7
So. magadii^*^	Soda soil, Magadi Lake, Kenya	11
So. tronii^*^	Soda soil, Magadi Lake, Kenya	
*Thielavia* sp.^*^	Lake of Holvo-Torum, Orongoyskoe, Russia; Yeraskhahun, Armenia	9.2–10.2
Verticillium zaregamsianum^*^	Trans-Baikal, Russia	7.6–8.2

* (Grum-Grzhimaylo et al., 2016);

# (Grum-Grzhimaylo et al., 2013a);

¶ (Nagai et al., 1995, 1998);

† (Georgieva et al., 2012);

‡ (Johnson, 1923);

♢ (Okada et al., 1993); (Liu et al., 2009);

$ (Zak and Wildman, 2004);

◙ *(Horikoshi, 1991)*.

Being an active decomposer fungi are the crucial component of carbon cycling and play active role in global climate change and carbon sequestration (Thormann, 2006). Fungal extracellular phenol oxidase/peroxidase is an important class of enzyme due to its participation in the degradation of lignin and toxic phenolics. The ABTS is a commonly used substrate to detect the activity of phenol oxidase (PO) in biological samples due to rapid oxidation of ABTS in colored blue-green ABTS^+^ by phenol oxidase. Although several different species of fungi with extracellular phenol oxidase producing potential have been isolated and characterized from different habitats (Szklarz et al., 1989; Crognale et al., 2012) but study on isolation and characterization of fungi with phenol oxidase producing potential from soda lake habitat are lacking. Isolation and characterization of phenol oxidase producing fungi from hyperalkaline and saline habitats like Lonar lake has special ecological significance because most of the contaminated habitat and industrial effluents have high alkalinity and salinity and fungal agent with survival potential in extreme condition with active phenol oxidase production potential can be used as bioinoculant for bioaugmentation based bioremediation. Furthermore, detection of active enzyme secretion in extreme conditions (high pH and salinity) indicates their probable role in the degradation of complex organics like leaf-litter, plant debris and detoxification of phenolics present in lake ecosystem and contribution in carbon turnover of the lake. Recently, Vavourakis et al. (2016) and Ausec et al. (2011) have shown in metagenomic studies of various soda lake that many uncultured fungi have laccases-like Cu-oxidase encoded which may be involved in degradation of phenolic compounds. Hence, this is the first culture based study on Soda lake showing phenolic compound degradation capacities of fungal isolates from such habitat.

Geographical location of Lonar lake (Figure 1) indicated that it is almost a closed ecosystem with only inflow of water by the surface run-off and discharge from a village effluent by small creek. It contains high levels of dissolved organic matter (DOM), high pH (pH 10), high salinity and high content of iron, magnesium and phosphorus which is generally considered not suitable for fungal growth. Despite unfavorable conditions for fungal growth, occurrence of wide range of fungi from diverse genera with polyphyletic affiliations with upland fungal species indicated that fungal flora of the Lonar lake are not a real habitants of the lake ecosystem. They might have arrived to the lake from outside in the form of spores and fruiting bodies. After that they adopted and established themselves according to the geographical conditions of the lake during the course of evolution and contributing in the functionality of the lake ecosystem. The present study provides first hand information about the diversity of fungi from hyperalkaline and saline Lonar lake. It is the first extensive investigation of the fungal diversity of Lonar lake and their physiological and functional potential in the lake. It will be interesting to study in detail the functional role of these fungi in the ecophysiology of lake habitat and mechanism by which they are able to tolerate and survive the extreme environment. Moreover, in depth sampling of the lake over time and area would help to study the spatial and temporal diversity and their impact on the lake ecology.

## Author contributions

RoS and OP conceived the study and also involved in sample collection, isolation and MS writing. RaS did phylogenetic study, novel species identification, and MS writing. PG, MS, and YN did molecular and enzymatic work.

## Funding

This work was supported by the Department of Biotechnology, New Delhi, India (BT/PR10054/NDB/52/94/2007). Senior Research Associateship of RaS is supported by the Council of Scientific and Industrial Research, New Delhi, India (CSIR Pool No. 8766-A).

### Conflict of interest statement

The authors declare that the research was conducted in the absence of any commercial or financial relationships that could be construed as a potential conflict of interest.
