# Supplementary material for: Diversity and Distribution of Phenol Oxidase Producing Fungi from Soda Lake and Description of Curvularia lonarensis sp. nov
Source: Front Microbiol. 2016 Nov 22;7:1847. doi: 10.3389/fmicb.2016.01847 (PMC5118452; doi:10.3389/fmicb.2016.01847)
Supplement: Supplementary file 1 [file Table1.DOCX]

Table ST1 Composition of media used for the isolation of fungi from Lonar lake.

| **HK Medium 8A** | |
| --- | --- |
| L-asparagine | 0.1 g/l |
| K_2_HPO_4_ | 0.5 g/l |
| FeSO_4_ | 0.001 g/l |
| MgSO_4_ | 0.1 g/l |
| Sodium caseinate | 2 g/l |
| C_3_H_5_NaO_2_ | 4 g/l |
| Agar | 20 g/l |
| **HK Medium 13A** | |
| Glucose | 4 g/l |
| Yeast extract | 4 g/l |
| Malt extract | 10 g/l |
| CaCO_3_ | 2 g/l |
| Agar | 20 g/l |
| **HK Medium 16A** |  |
| Soya bean | 20 g/l |
| Mannitol | 20 g/l |
| Agar | 20 g/l |
| **HK Medium 19A** | |
| K_2_HPO_4_ | 1.77 g/l |
| KH_2_PO_4_ | 0.68 g/l |
| NaCl | 0.14 g/l |
| CaCl_2_ | 0.132 g/l |
| MgSO_4_ .7H_2_O | 0.2 g/l |
| Mannitol | 10 g/l |
| Yeast extract | 0.08 g/l |
| FeSO_4_.7H_2_O | 0.0025 g/l |
| H_3_BO_3_ | 0.0029 g/l |
| CoSO_4_.7H_2_O | 0.0012 g/l |
| CuSO_4_.5H_2_O | 0.0001 g/l |
| MgCl_2_.4H_2_O | 0.00009 g/l |
| Na_2_MoO_4_.2H_2_O | 0.0025 g/l |
| ZnSO_4_.7H_2_O | 0.0012 g/l |
| Agar | 20 g/l |
| **HK Medium 21A** | |
| Casein enzymic hydrolysate | 5 g/l |
| Yeast extract | 1 g/l |
| Agar | 20 g/l |
| **HK Medium 22A** | |
| Tryptone | 10 g/l |
| Proteose peptone | 10 g/l |
| K_2_HPO_4_ | 1.5 g/l |
| MgSO_4_ | 1.5 g/l |
| Agar | 20 g/l |
| **HK Medium 28A** | |
| Potassium phosphate | 15 g/l |
| (NH_4_)_2_SO_4_ | 1 g/l |
| MgSO_4_ | 0.2 g/l |
| CaCl_2_ | 0.01 g/l |
| Infusion broth | 25 g/l |
| Dextrose | 5 g/l |
| L-cysteine hydrochloride | 1 g/l |
| Pancreatic digest of casein | 4 g/l |
| Yeast extract | 5 g/l |
| Soluble starch | 1 g/l |
| Agar | 20 g/l |
