# Supplementary material for: Diversity and Distribution of Phenol Oxidase Producing Fungi from Soda Lake and Description of Curvularia lonarensis sp. nov
Source: Front Microbiol. 2016 Nov 22;7:1847. doi: 10.3389/fmicb.2016.01847 (PMC5118452; doi:10.3389/fmicb.2016.01847)
Supplement: Supplementary file 2 [file Table2.DOCX]

Table ST2GenBank accession no., strain or culture collection no. and source of isolates included in the phylogenetic study.

| **Species** | **Strain No.** | **Source** | **ITS** | **LSU** | ***gpd*** |
| --- | --- | --- | --- | --- | --- |
| *Bipolaris chloridis* | CBS 242.77B | *Chloris gayana*, Australia | HF934928 | HF934869 | HG779083 |
| *B. cynodontis* | CBS 285.51 | *Cynodon transvaalensis*, Kenya | HF934929 | HF934874 | HG779081 |
| *B. cynodontis* | CBS 305.64 | *Cynodon dactylon*, USA | HF934930 | HF934883 | HG779082 |
| *B. maydis* | CBS 136.29 | *Zea mays*, Japan | HF934926 | HF934879 | HG779086 |
| *B. maydis* | CBS 307.64 | *Zea mays*, USA | HF934925 | HF934875 | HG779085 |
| *B. microlaenae* | CBS 280.91^T^ | *Microlaena stipoides* leaf, Australia | HF934933 | HF934877 | HG779092 |
| *B. oryzae* | CBS 157.50 | *Oryza sativa* grain, Indonesia | HF934931 | HF934870 | HG779090 |
| *B. oryzae* | CBS 199.54 | *Oryza sativa* grain, New Guinea | HF934932 | HF934884 | HG779091 |
| *B. sorghicola* | CBS 249.49 | *Sorghum vulgare* var. *sudanense*, Locality unknown | HF934927 | HF934868 | HG779087 |
| *B. sorokiniana* | CBS 140.31 | Substrate unknown, Japan | HF934935 | HF934876 | HG779088 |
| *B. sorokiniana* | CBS 145.32 | *Triticum durum*, Locality unknown | HF934934 | HF934885 | HG779089 |
| *B. zeae* | CBS 127716 | Unknown | HG778980 | HG779027 | HG779095 |
| *B. zeicola* | CBS 316.64 | *Zea mays*, USA | HF934938 | HF934871 | HG779093 |
| *B. zeicola* | CBS 317.64 | *Zea mays*, USA | HF934939 | HF934878 | HG779094 |
| *Curvularia aeria* | CBS 294.61^T^ | Air, Brazil | HF934910 | HF934902 | HF565450 |
| *C. affinis* | CBS 154.34^T^ | *Manihot utilissima*, Java | HG778981 | HG779028 | HG779126 |
| *C. affinis* | CBS 185.49 | *Manihot utilissima*, Java | HG778982 | HG779029 | HG779127 |
| *C. akaii* | CBS 318.86 | Substrate unknown, Japan | HF934921 | HF934897 | HG779118 |
| *C. akaii* | CBS 127730 | Substrate unknown, Japan | HF934922 | HF934899 | HG779120 |
| *C. americana* | UTHSC 08-3414^T^ | Ankle, USA | HE861833 | HG779056 | HF565488 |
| *C. americana* | UTHSC 07-2649 | Toe tissue, USA | HE861834 | HG779054 | HF565486 |
| *C. australiensis* | CBS 172.57 | *Oryza sativa* seed, Vietnam | HF934912 | HF934901 | HG779139 |
| *C. australis* | BRIP 12525 | Australia | AF081448 | — | AF081409 |
| *C. brachyspora* | CBS 186.50 | Soil, Java | HG778983 | HG779030 | HG779150 |
| *C. carica-papayae* | CBS 135941T | Carica papaya leaf, India | HG778984 | HG779031 | HG779146 |
| *C. chlamydospora* | UTHSC 07-2764^T^ | Toe nail, USA | HG779021 | HG779075 | HG779151 |
| *C. chlamydospora* | UTHSC 08-1283 | Nasal sinus, USA | HG779022 | HG779076 | HG779152 |
| *C. coicis* | CBS 192.29^T^ | *Coix lacrima*-*jobi* var. *typica*, Japan | HF934917 | HF934895 | HG779130 |
| *C. cymbopogonis* | CBS 419.78 | *Yucca* sp. leaf, Netherlands | HG778985 | HG779032 | HG779129 |
| *C. dactyloctenii* | BRIP 12913 | Australia | AF071322 | — | AF081376 |
| *C. ellisii* | CBS 193.62 | Air, Pakistan | HF934913 | HF934896 | HG779143 |
| *C. eragrostidis* | CBS 189.48 | Sorghum seed, Java | HG778986 | HG779033 | HG779154 |
| *C. geniculata* | CBS 187.50 | Indonesia | KJ909781 | KM243260 | KM083609 |
| *C. gladioli* | CBS 210.79 | *Gladiolus* sp. leaf, Romania | HG778987 | HG779034 | HG779123 |
| *C. graminicola* | BRIP 23186 | *Aristida ingrata*, Australia | JN192376 | JN600986 | JN600964 |
| *C. hawaiiensis* | CBS 173.57^T^ | *Oryza sativa*, Hawaii | HG778988 | HG779035 | HG779140 |
| *C. hawaiiensis* | CBS 448.72 | Salt-marsh soil, Kuwait | HG778989 | HG779036 | HG779142 |
| *C. heteropogonis* | CBS 284.91T | *Heteropogon contortus* leaf, Australia | HF934919 | HF934893 | JN600969 |
| *C. heteropogonis* | CBS 511.91 | *Heteropogon contortus* leaf, Australia | HF934918 | HF934894 | HG779122 |
| *C. hominis* | UTHSC 09-464^T^ | Cornea, USA | HG779011 | HG779065 | HG779106 |
| *C. hominis* | UTHSC 08-2517 | Foot, USA | HG779009 | HG779063 | HG779107 |
| *C. homomorpha* | DAOM 63822 | USA | KM257055 | — | KM257058 |
| *C. inaequalis* | CBS 102.42 | France | KJ922375 | KM243261 | KM061787 |
| *C. intermedia* | CBS 334.64 | *Avena versicolor*, USA | HG778991 | HG779038 | HG779155 |
| *C. ischaemi* | CBS 630.82^T^ | *Ischaemum indicum* leaf, Solomon Islands | HG778992 | HG779039 | HG779131 |
| *C. kusonoi* | CBS 137.29 | Japan | JN192381 | JN600993 | — |
| *C. lonarensis sp. nov.* | CBS 140569^T^ = MCC 1209^T^ | Lonar lake, India | KTT315408 | KY007018 | KY007019 |
| *C. lunata* | CBS 730.96^NT^ | Lung biopsy, USA | HF934911 | HF934900 | KC485081 |
| *C. miyakei* | CBS 197.29 | Japan | KJ909770 | — | KM083611 |
| *C. muehlenbeckiae* | CBS 144.63^T^ | *Muehlenbeckia* sp. leaf, India | HG779002 | HG779049 | HG779108 |
| *C. muehlenbeckiae* | UTHSC 08-2905 | Chest, USA | HE861836 | HG779050 | HF565484 |
| *C. neergaardii* | DAOM 228085 | Chile | KJ909784 | — | KM083615 |
| *C. neoindica* | BRIP 17439 | Australia | AF081449 | — | AF081406 |
| *C. nicotiae* | CBS 655.74 | Algeria | KJ909772 | KM243291 | KM083614 |
| *C. nodulosa* | CBS 160.58 | USA | JN601033 | JN600997 | JN600975 |
| *C. oryzae* | CBS 169.53^T^ | *Oryza sativa* seed, Vietnam | HF934906 | HF934867 | HG779156 |
| *C. ovariicola* | CBS 285.91 | *Eragrostis parviflora*, Australia | HG778993 | HG779040 | HG779144 |
| *C. ovariicola* | CBS 286.91 | *Eragrostis parviflora*, Australia | HG778994 | HG779041 | HG779145 |
| *C. pallescens* | CBS 156.35 | Java | KJ922380 | KM243269 | KM083606 |
| *C. papendorfii* | CBS 308.67 | South Africa | KJ909774 | KM243290 | KM083617 |
| *C. perotidis* | CBS 350.90^T^ | *Perotis rara*, Australia | HG778995 | HG779042 | HG779138 |
| *C. portulacae* | CBS 239.48 | USA | KJ909775 | KM243292 | KM083616 |
| *C. prasadii* | CBS 143.64^T^ | *Jasminum sambac*, India | HG778996 | HG779043 | HG779147 |
| *C. prasadii* | CBS 144.64 | Substrate unknown, England | HG778997 | HG779044 | HG779149 |
| *C. protuberata* | CBS 376.65^T^ | *Deschampsia flexuosa* leaf, Scotland | HG778998 | HG779045 | HG779135 |
| *C. ravenelii* | CBS 127709 | Unknown | HG778999 | HG779046 | HG779109 |
| *C. robusta* | CBS 624.68^T^ | *Dichanthium annulatum* leaf, USA | HG779000 | HG779047 | HG779125 |
| *C. ryleyi* | CBS 349.90 | Australia | KJ909766 | KM243267 | KM083612 |
| *C. senegalensis* | CBS 149.71 | Substrate unknown, Nigeria | HG779001 | HG779048 | HG779128 |
| *C. sesuvii* | BpZj 01 | China | EF175940 | — | — |
| *C. spicifera* | CBS 198.31 | *Capsicum anuum*, Cyprus | HF934916 | HF934905 | HG779136 |
| *C. spicifera* | CBS 199.31 | *Cucurbita maxima*, Cyprus | HF934915 | HF934903 | HG779137 |
| *C. subpapendorfii* | CBS 656.74 | Egypt | KJ909777 | KM243266 | KM061791 |
| *C. trifolii* | CBS 173.55 | *Trifolium repens*, USA | HG779023 | HG779077 | HG779124 |
| *C. tripogonis* | BRIP 12375^T^ | *Tripogon jacquemonti*, India | JN192388 | JN601002 | JN600980 |
| *C. tuberculata* | CBS 146.63^T^ | *Zea mays* leaf, India | HF934907 | HF934866 | HG779157 |
| *C. uncinata* | CBS 221.52^T^ | *Oryza sativa* leaf, Vietnam | HG779024 | HG779078 | HG779134 |
| *C. uncinata* | CBS 531.70 | *Oryza sativa* seeds, Denmark | HG779025 | HG779079 | HG779132 |
| *C. verruciformis* | CBS 537.75 | *Lobibyx* sp. feather, New Zealand | HG779026 | HG779080 | HG779133 |
| *C. verruculosa* | CBS 149.63 | *Elaeis guineensis*, Nigeria | HF934909 | HF934891 | HG779110 |
| *C. verruculosa* | CBS 150.63 | *Punica granatum* leaf, India | HF934908 | HF934892 | HG779111 |
| *Setosphaeria turcica* | CBS 330.64 | *Zea mays*, USA | HF934950 | HF934887 | HG779153 |
