# Supplementary figures and images for: Diversity and Distribution of Phenol Oxidase Producing Fungi from Soda Lake and Description of Curvularia lonarensis sp. nov
Source: Front Microbiol. 2016 Nov 22;7:1847. doi: 10.3389/fmicb.2016.01847 (PMC5118452; doi:10.3389/fmicb.2016.01847)

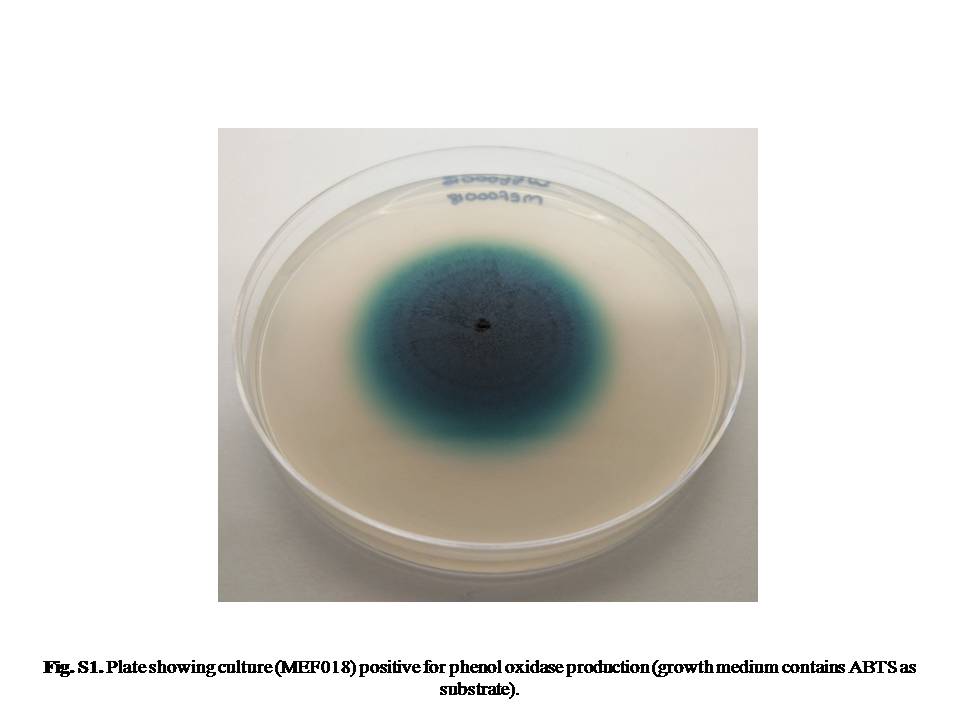

Supplement: Supplementary file 3 [file Image1.JPEG]

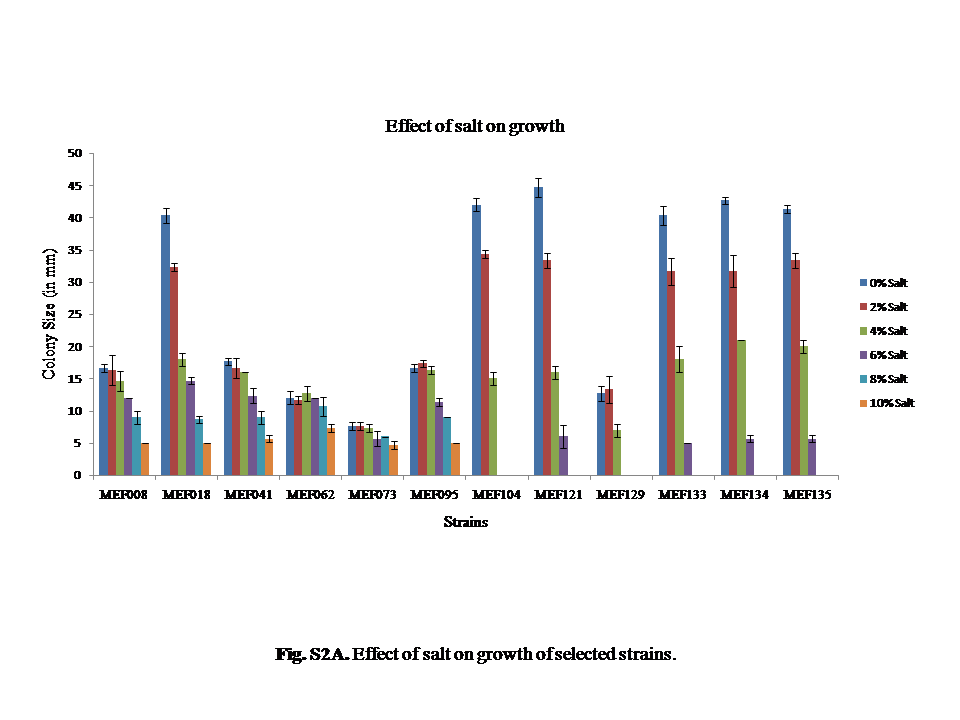

Supplement: Supplementary file 4 [file Image2.TIF]

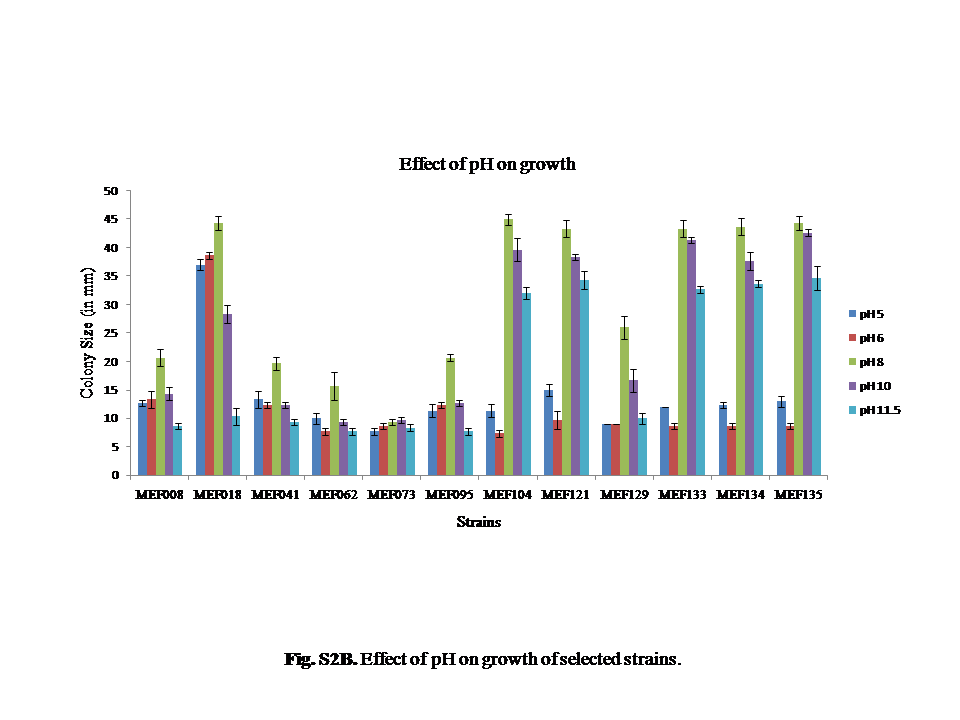

Supplement: Supplementary file 5 [file Image3.TIF]

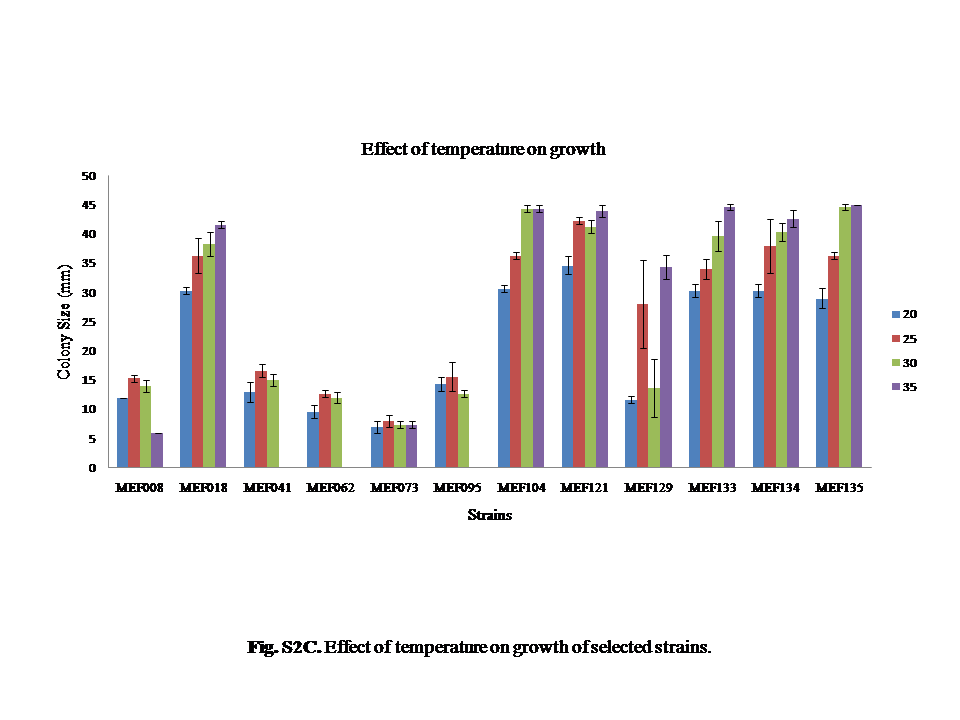

Supplement: Supplementary file 6 [file Image4.TIF]

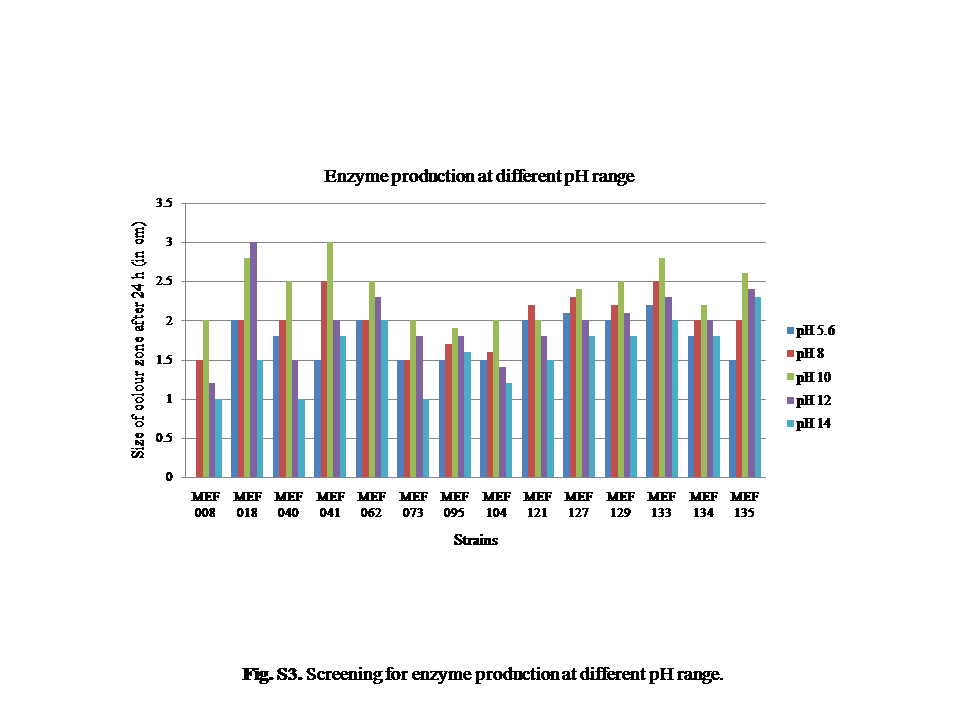

Supplement: Supplementary file 7 [file Image5.TIF]

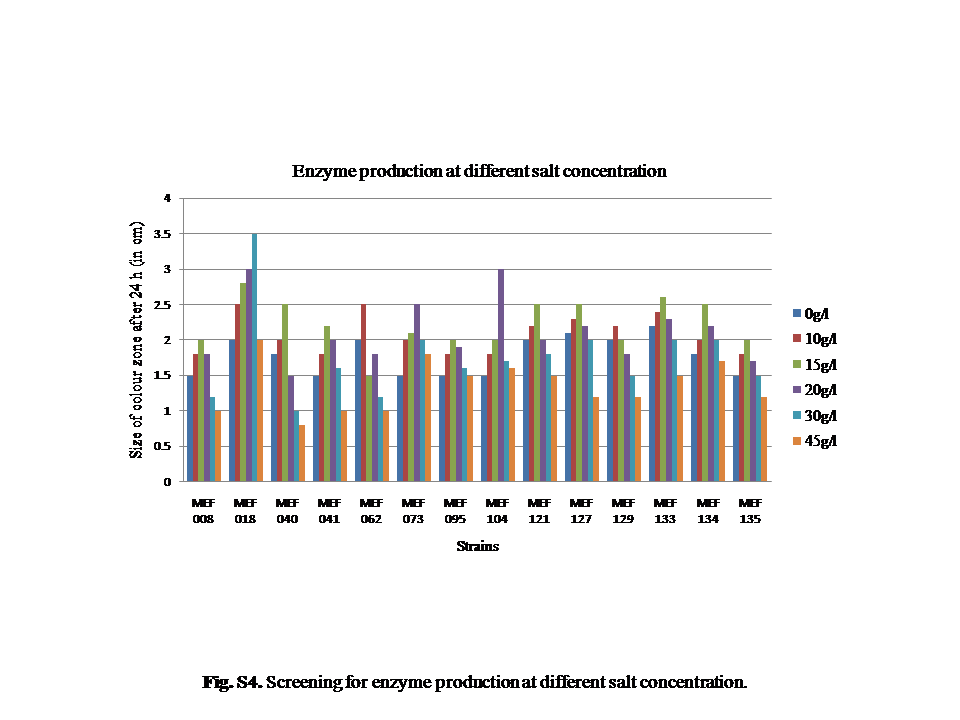

Supplement: Supplementary file 8 [file Image6.TIF]

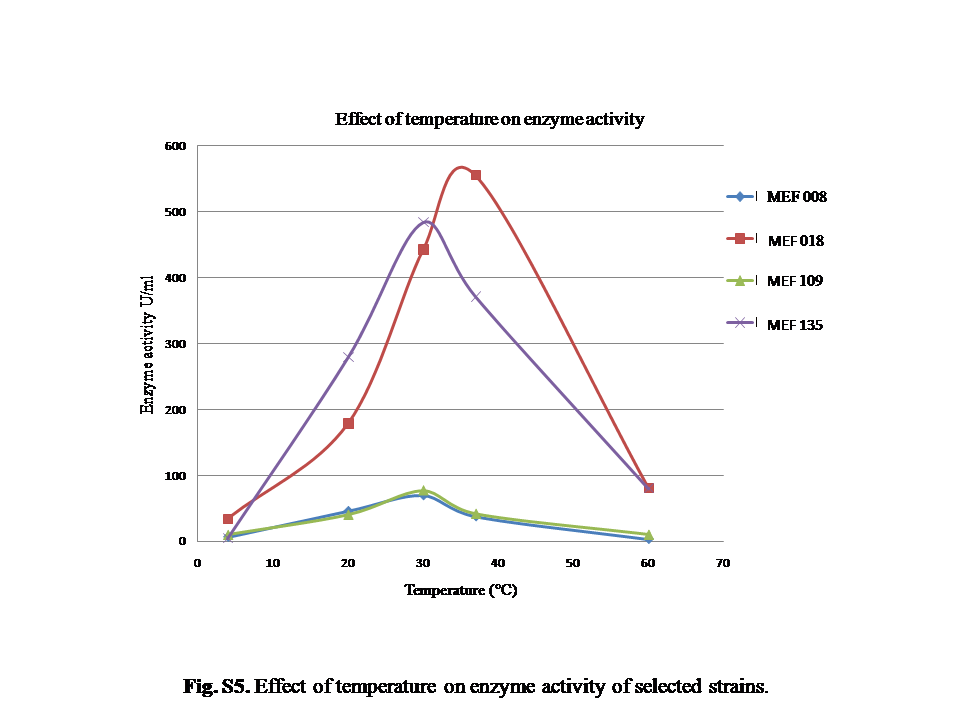

Supplement: Supplementary file 9 [file Image7.TIF]

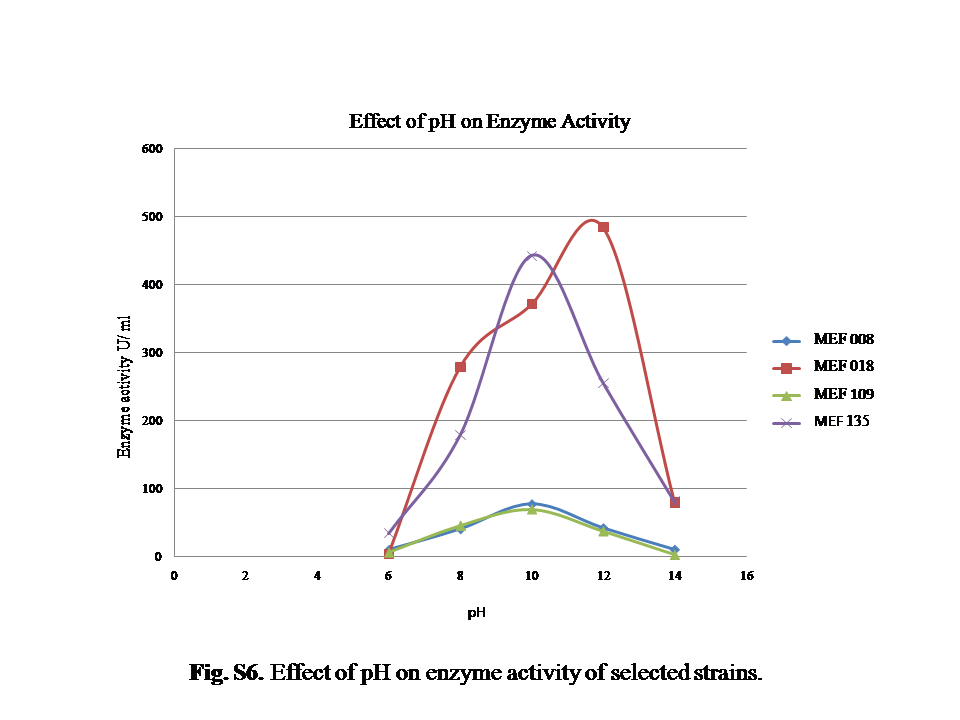

Supplement: Supplementary file 10 [file Image8.TIF]

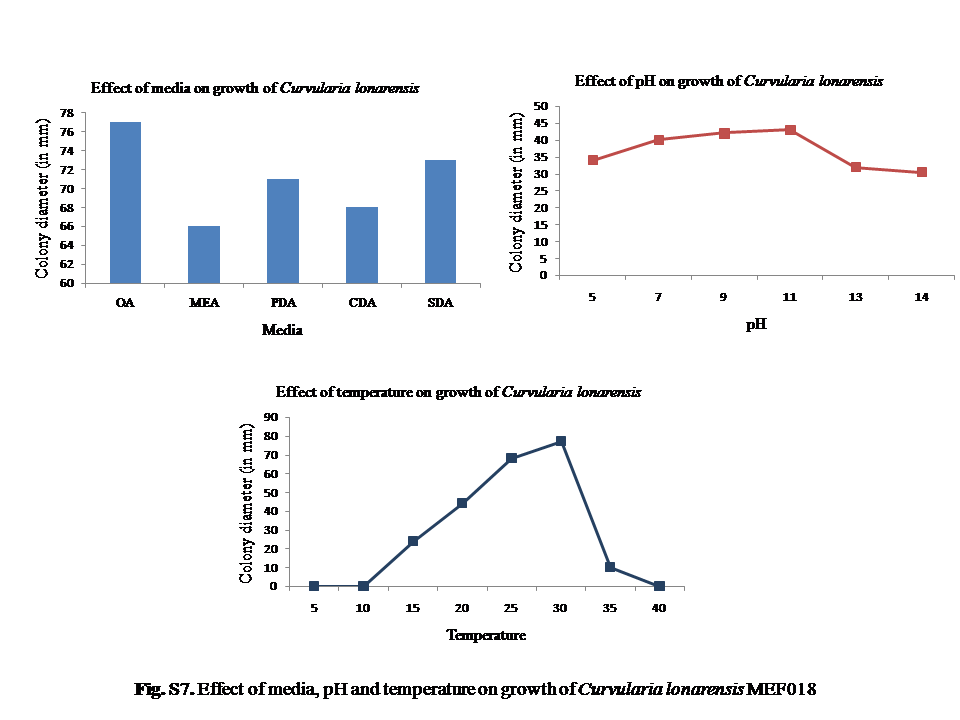

Supplement: Supplementary file 11 [file Image9.TIF]
